# MicroRNA-guided prioritization of genome-wide association signals reveals the importance of microRNA-target gene networks for complex traits in cattle

**DOI:** 10.1038/s41598-018-27729-y

**Published:** 2018-06-19

**Authors:** Lingzhao Fang, Peter Sørensen, Goutam Sahana, Frank Panitz, Guosheng Su, Shengli Zhang, Ying Yu, Bingjie Li, Li Ma, George Liu, Mogens Sandø Lund, Bo Thomsen

**Affiliations:** 1 Center for Quantitative Genetics and Genomics, Department of Molecular Biology and Genetics, Aarhus University, 8830 Tjele, Denmark; 2 Section for Molecular Genetics and Systems Biology, Department of Molecular Biology and Genetics, Aarhus University, 8000 Aarhus C, Denmark; 3 Key Laboratory of Animal Genetics, Breeding and Reproduction, Ministry of Agriculture & National Engineering Laboratory for Animal Breeding, College of Animal Science and Technology, China Agricultural University, 100193 Beijing, China; 4 Department of Animal and Avian Sciences, University of Maryland, College Park, 20742 MD USA; 5 Animal Genomics and Improvement Laboratory, ARS USDA, Beltsville, 207052350 MD USA

## Abstract

MicroRNAs (miRNA) are key modulators of gene expression and so act as putative fine-tuners of complex phenotypes. Here, we hypothesized that causal variants of complex traits are enriched in miRNAs and miRNA-target networks. First, we conducted a genome-wide association study (GWAS) for seven functional and milk production traits using imputed sequence variants (13~15 million) and >10,000 animals from three dairy cattle breeds, *i*.*e*., Holstein (HOL), Nordic red cattle (RDC) and Jersey (JER). Second, we analyzed for enrichments of association signals in miRNAs and their miRNA-target networks. Our results demonstrated that genomic regions harboring miRNA genes were significantly (P < 0.05) enriched with GWAS signals for milk production traits and mastitis, and that enrichments within miRNA-target gene networks were significantly higher than in random gene-sets for the majority of traits. Furthermore, most between-trait and across-breed correlations of enrichments with miRNA-target networks were significantly greater than with random gene-sets, suggesting pleiotropic effects of miRNAs. Intriguingly, genes that were differentially expressed in response to mammary gland infections were significantly enriched in the miRNA-target networks associated with mastitis. All these findings were consistent across three breeds. Collectively, our observations demonstrate the importance of miRNAs and their targets for the expression of complex traits.

## Introduction

Understanding the genetic architecture, *i*.*e*., knowledge of causal genomic variants, their allele frequencies and effect sizes, underpinning complex phenotypes and diseases is a long ongoing quest in the field of genetics and genomics^1–3^. Complex phenotypes consist of contributions from multiple genomic loci, and accurate and reliable prediction of future complex traits and diseases based on genomic information is critical for human personalized medicine as well as for breeding of agricultural animal and plant species^4–11^. In the past decade, genome-wide association studies (GWAS) have been a productive way to achieve this goal^5^. Yet, big challenges remained in extending GWAS results to informative biological hypotheses underlying complex traits variation primarily due to 1) poor detection of many causal loci/genes with small effects by GWAS with limited sample sizes^12,13^, and 2) masking of true association signals by linkage disequilibrium (LD) between causal loci and neighboring markers^4,14^, especially in livestock with high degree of relatedness^10,15^. Recently, we have shown that the predictive ability of the genomic best linear unbiased prediction model increases in accuracy when the model utilizes and quantifies the combined contributions of markers in genomic regions associated with the genetic architecture of the underlying trait^9,16–18^. Also, biological knowledge has been incorporated to improve the understanding of GWAS results^19–25^. For instance, by integrating genome functional annotations with GWAS data, Finucane *et al*. (2015) revealed conserved genomic regions that were strongly enriched with genetic variation of many complex traits in human^25^. Holmans *et al*. (2009) provided insights into the biology of bipolar disorder through incorporation of Gene Ontology (GO) annotation into GWAS results^23^. Fang *et al*. (2017) revealed novel insights into the genetic and biological basis underpinning mastitis by incorporating transcriptome data^22^.

GWAS signals often map to non-coding regions of the genome, demonstrating that sequence variants in protein coding genes alone cannot explain most quantitative phenotypes. Indeed, conventional protein coding genes constitute only a very small percentage of the genome; yet ~75% of the genome generates RNA transcripts^26^. Thus, most transcripts in eukaryotic cells are non-coding RNA, and much of the regulatory capability and function of the genome are provided by non-coding RNA transcripts^26–28^. Among the many classes of non-coding RNA species are microRNAs, which act posttranscriptionally to fine-tune protein synthesis. The vertebrate genome encodes in the order of several hundred microRNAs comprising both evolutionary conserved and species-specific genes. The encoded miRNAs are ~22 nucleotide long RNA molecules that align to sequences in target mRNA molecules, leading to either deadenylation and decay of mRNAs or repression of protein translation^29^. In more rare cases, miRNAs can also activate and up-regulate gene expression in presence of distinct cofactors and under specific cellular conditions^30^. A single miRNA can target several different mRNAs, and moreover, mRNAs are often controlled by several different miRNAs^29^. Computational estimates suggest that more than 30% of all protein-coding genes in human are regulated by miRNAs^31–34^. This strongly indicates that miRNAs have widespread roles *in vivo*, and that all genetic networks and pathways are expected to be regulated to some degree by miRNAs. Genomic sequence variation can affect miRNAs functions in several ways: Variants can occur in sequences that are responsible for driving the expression of miRNA genes; variants can disrupt or create miRNA binding sites in target mRNAs; and the miRNAs themselves can exist as variants^35^. In this study, we aimed to investigate the joint effect of genetic variations in miRNA genes and in their targets-networks on complex traits of economic importance in dairy cattle.

## Results

**Figure 1:**
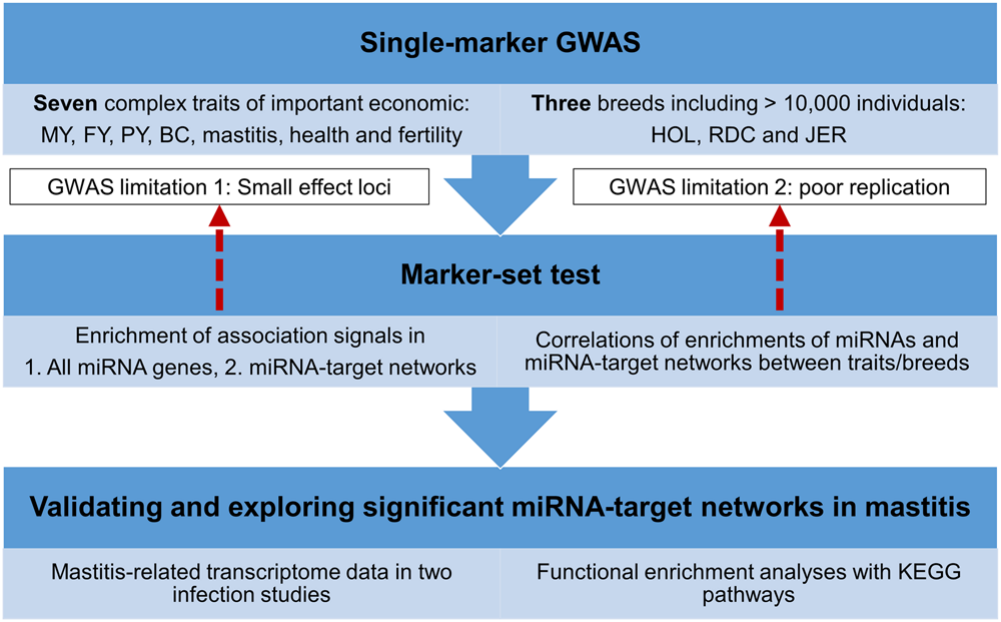
The overall study design. MY, FY, PY and BC are milk yield, fat yield, protein yield, and body conformation, respectively. HOL, RDC, and JER are Holstein, Nordic red, and Jersey cattle.

The overall study design is shown in Fig. 1. The quantitative traits in this study were body conformation (BC), mastitis, health, fertility and three milk production traits, including fat yield (FY), milk yield (MY) and protein yield (PY). Index for general health in dairy cattle describes genetic potential to resist reproductive, metabolic and feet-and-leg diseases, and this index is calculated based on data of veterinarians’ treatments in the first three lactations (http://www.nordicebv.info). We used imputed sequence variants (13~15 million single-nucleotide polymorphism (SNPs)) to ensure the coverage of most genes and miRNAs in the bovine genome (UMD 3.1). We performed within breed analyses for Holstein (HOL, n = 5,056), Nordic red (RDC, n = 4,310) and Jersey (JER, n = 1,231) cattle, to confirm our findings. Infection-induced transcriptome data were then integrated to validate the significant miRNA-target networks detected for mastitis, and to investigate the underlying genetic and biological basis of our findings.

### Single-marker GWAS for seven quantitative traits in three cattle breeds

The single-marker GWAS was performed to identify genomic variants associated with the traits being studied. The −log_10_*P* values of all tested sequence variants in the seven traits across HOL, RDC and JER are shown in the Manhattan plots, Supplementary Fig. S1, S2 and S3, respectively. At a nominal *P*-value < 3.3 × 10^−9^ (Bonferroni correction), a total of 6, 4, 12, 0, 5, 1 and 2 quantitative trait loci (QTL) were detected for MY, FY, PY, BC, mastitis, health and fertility, respectively in HOL, which jointly explained 24.1%, 22.3%, 19.3%, 0%, 9.7%, 8.2% and 5.3% of genomic variance in each corresponding trait, respectively. In RDC with the same *P*-value threshold as in HOL, 6, 6, 6, 4, 2, 0 and 4 QTLs were detected for MY, FY, PY, BC, mastitis, health and fertility respectively, which jointly explained 13.0%, 13.3%, 9.3%, 33.3%, 6.8%, 0% and 8.1% of genomic variance for each trait, respectively. In JER at *P*-value < 3.7 × 10^−9^, only 2 and 1 QTLs were detected for MY and FY, respectively, accounting for 3.9% and 3.1% of genomic variance for each trait, respectively. All the results demonstrated that the detected QTLs explained only a small fraction of genomic variance in complex traits, particularly in JER with a small sample size. The details of QTLs for all seven traits in the three breeds are summarized in Supplementary Table S1.

### MiRNA genes are enriched with GWAS signals

**Figure 2:**
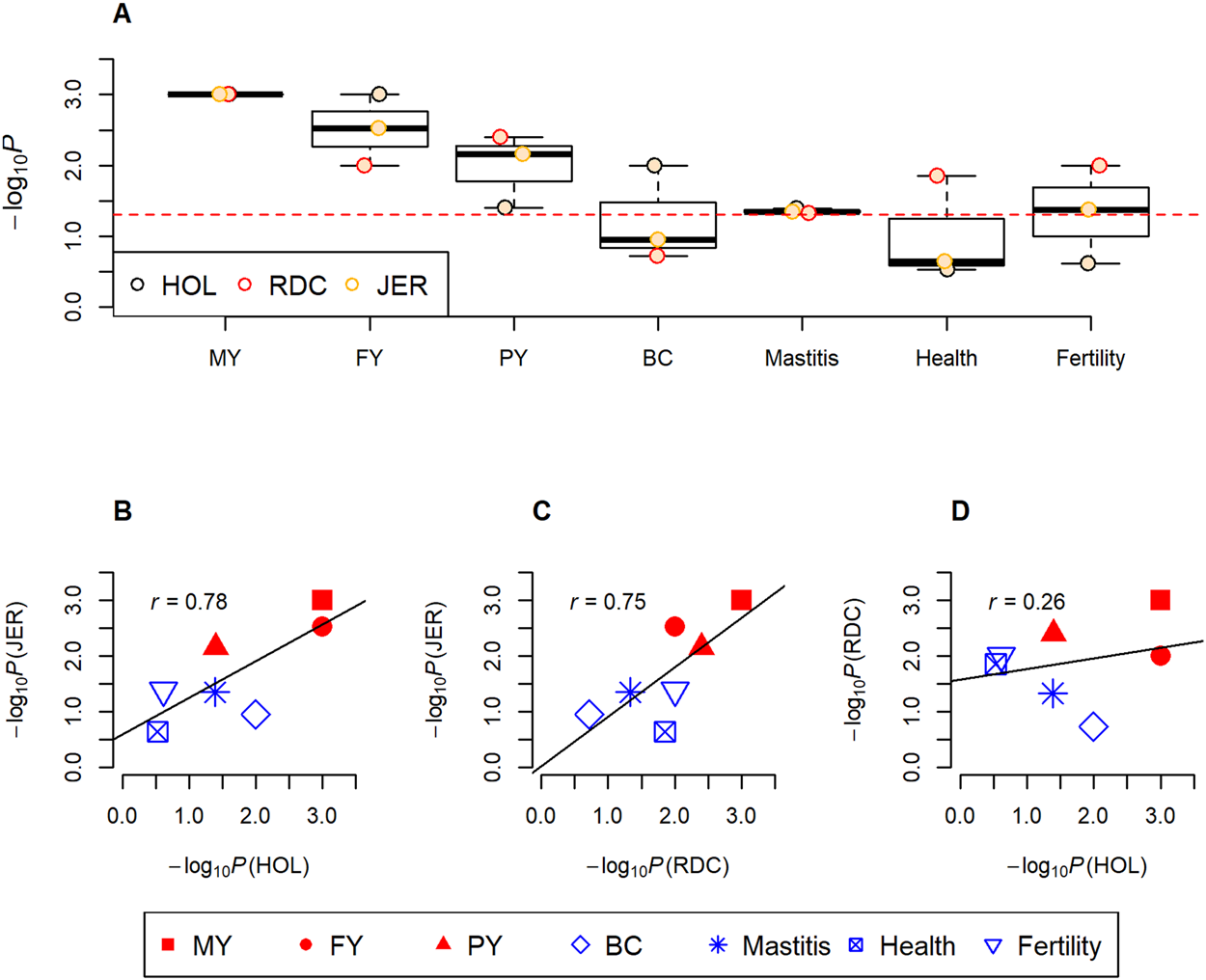
The enrichments (*i*.*e*., −log_10_*P*) of association signals in miRNA genes with ±5 kb extension for seven traits across three breeds. (**A**) is the enrichments for seven traits among three breeds; the red dotted line corresponds to *P* = 0.05. (**B**–**D**) are correlations of enrichments for all seven traits in Holstein (HOL) *vs*. Jersey (JER), Nordic red cattle (RDC) *vs*. JER, and HOL *vs*. RDC, respectively. MY, FY, PY and BC are milk yield, fat yield, protein yield, and body conformation, respectively. −log_10_*P* is determined by marker-set test.

In order to assess whether miRNAs influence the traits, we first mapped known miRNA genes in the bovine genome (UMD 3.1). A total of 750 bovine autosome miRNA genes expressed in different tissues from miRbase^36^ (http://www.mirbase.org/ftp.shtml) were observed to be distributed with an average of 26 (range: 7 to 77) miRNAs per chromosome (Fig. S4A and B). The average lengths of the miRNA precursor sequences were 77 base-pairs (bp) (range: 52 to 148 bp) (Fig. S4C). Since very few imputed SNPs were observed within the miRNA precursor regions due to their short lengths (see Supplementary Table S2), the analysis included the flanking ±3 kb, ±5 kb, ±0 kb, ±20 kb, ±50 kb sequences of all miRNA genes to capture proximal SNPs in the regulatory regions. Details of miRNAs and SNPs involved in the analyses under each extension are shown in Supplementary Table S1. Considering all miRNA genes together as a single set of markers, the marker-set test was carried out to analyze for enrichment of miRNA genes with association signals for each trait in all breeds. Analysis using the miRNA ±5 kb extended regions, significant (*P* < 0.05) enrichments were observed for most traits in the three breeds, especially for three milk production traits and mastitis (Fig. 2A), and the enrichments (*i*.*e*., −log_10_*P* values, marker-set test) were correlated between breeds (Fig. 2B–D). Of note, after removing all the four miRNAs (*i*.*e*., bta-miR-2309, bta-miR-1839, bta-miR-2308, and bta-miR-193a-2) that were in close proximity to the well-known milk/fat gene, *DGAT1*, on *Bos taurus* chromosome 14 (BTA 14)^37^, similar enrichments were still observed for the three production traits across the three breeds, particularly in RDC and JER (see Supplementary Table S3). In addition, similar enrichments were observed with other extensions for all seven traits in three breeds due to the extensive LD in bovine genome^15^ (see Supplementary Fig. S5). These results suggested that the phenotypic variations in the traits may be associated with the miRNA genes.

### MiRNA-target networks provide new insights into the genetic architecture of complex traits

**Figure 3:**
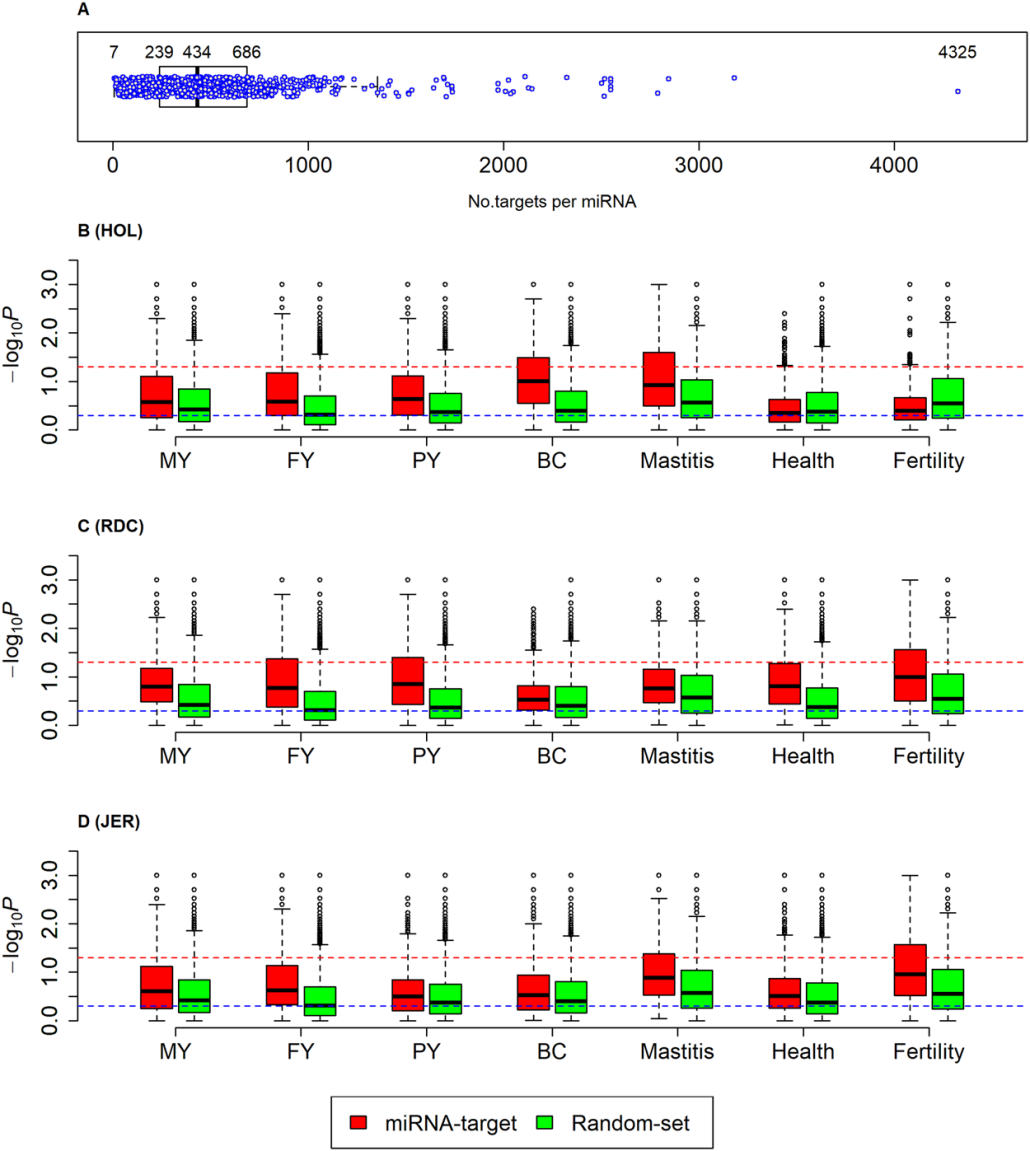
Number of targets of miRNAs and the enrichments with miRNA-target networks for seven traits across three breeds. (**A**) Shows the number of target genes in each miRNA, and each blue point is a single miRNA. (**B**–**D**) are the difference in enrichments (*i*.*e*., −log_10_*P*) between the miRNA-target networks (miRNA-target) and random-target networks (Random-set) for seven traits in Holstein (HOL), Nordic red cattle (RDC), and Jersey (JER), respectively. Random-set is the random target-networks of miRNAs (replicates = 1000). −log_10_*P* is calculated using marker-set test. MY, FY, PY and BC are milk yield, fat yield, protein yield, and body conformation, respectively. The red dotted line corresponds to *P* = 0.05, and the blue line corresponds to *P* = 0.5.

The targets of trait-associated miRNAs are likely to be enriched for GWAS signals. To test this, we employed the miRmap software to *in silico* predict targets of all miRNAs^38^ (http://mirmap.ezlab.org/). Computational target prediction remains a challenge as they predicted targets only rely on the physical properties of miRNA regulation (e.g., evolutionary conservation and secondary structures of 3′ UTRs), hence only the top 25% of predicted targets were analyzed in the downstream analysis, including 11,455 out of 24,616 bovine genes (UMD 3.1, http://www.ensembl.org/Bos_taurus/Info/Index). The average number of targets of miRNAs was 434 (range: 7 to 4,325) (Fig. 3A). To test for enrichment of GWAS signals, all targets of a single miRNA were considered as a miRNA target-network (*i*.*e*., as one marker set) and only SNPs located in the open reading frame (ORF) of targets were included. Furthermore, for comparison, 1,000 random target networks were generated for each miRNA, and 769 GO terms together with 916 Reactome terms were also analyzed (see Methods).

Compared to the random-target networks, the enrichments with miRNA-target networks were significantly (*P* < 0.05, Wilcoxon-test) higher for all seven traits across three breeds, except for health and fertility in HOL (Fig. 3B–D). This was in agreement with the finding that fertility and health in HOL had the lowest enrichments with miRNA genes themselves as shown in Fig. 3A. Furthermore, the enrichments of fertility and health in both RDC and JER were significantly higher than in HOL, and the enrichment of BC in HOL was significantly higher than in RDC and JER, which were also in line with that the observations on the miRNA genes.

### Correlations between traits

**Figure 4:**
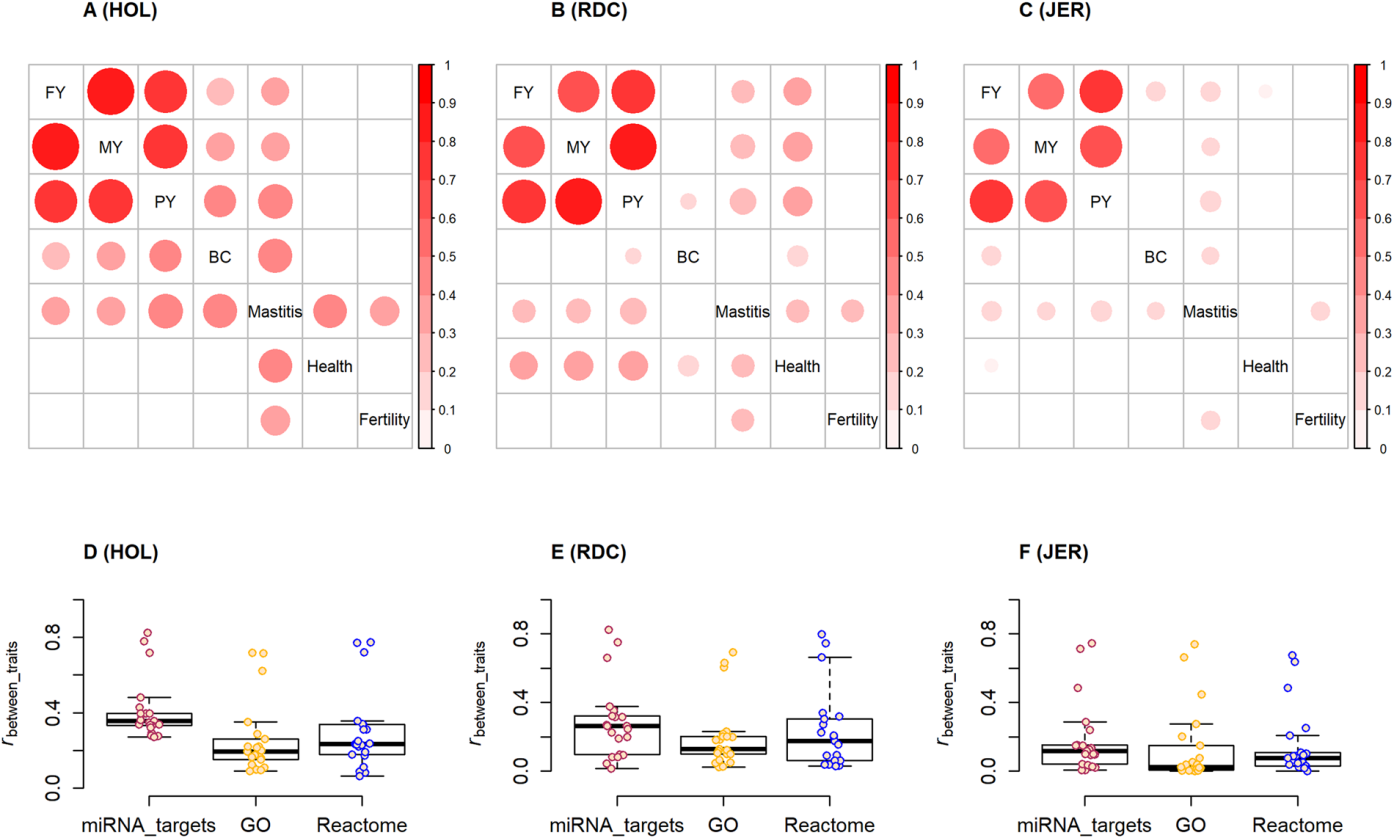
The correlations for enrichments with miRNA-targets networks between traits in Holstein (HOL), Nordic red cattle (RDC) and Jersey (JER). (**A**–**C**) are the singificant (*P* < 0.05) between-trait correlations in HOL, RDC, and JER, respectively. The significant level was computed by assumping the correlation follows a normal distribution that was determined by random-target networks (replicates = 1,000). (**D**–**F**) are the difference in between-trait correlations among miRNA-target networks, Gene Ontology (GO) and Reactome terms for HOL, RDC, and JER, respectively.

The enrichments with miRNA-target networks were highly significantly (*P* < 0.001) correlated between traits in HOL, and the highest correlations were observed among milk production traits (see Supplementary Fig. S6). Similar patterns were also observed in both RDC and JER, except for health and fertility in JER which might be due to their low statistical power (*i*.*e*., the combined influence of the small sample size and the small effect size) in GWAS (see Supplementary Figs S7 and S8). Assuming each correlation coefficient approximately follows a normal distribution, which was determined by 1,000 random-target networks (see Methods), a majority of between-traits correlations with miRNA-target networks were significantly (*P* < 0.05) greater than the random correlation coefficients, demonstrating that the genetic architecture of multiple complex traits shared certain similarities in the underlying miRNA-target networks. Only the significant between-traits correlations in HOL, RDC and JER are shown in Fig. 4A–C, respectively. The correlations among milk production traits were significant across three breeds. The correlations between BC with all three milk production traits were significant in HOL, and the correlations between health with other six traits (expect for BC) were significant in RDC. Of special note, mastitis was significantly correlated with the remaining six traits across all three breeds, except for mastitis *vs*. BC in RDC, and mastitis *vs*. health in JER. Additionally, the between-trait correlations with miRNA-target networks were typically higher than those with GO and Reactome terms, and the patterns were consistent across the three breeds (Fig. 4D–F). All the findings here provided genomic evidence that miRNAs may influence many complex phenotypes through regulation of their targets, and further suggests pleiotropic effects of miRNAs.

### Correlations between breeds

The correlations between breeds of enrichments with miRNA-target networks for the same trait were calculated. As shown in Table 1, a majority of between-breed correlations were significantly greater than the random correlation coefficients (*P* < 0.05). Furthermore, the between-breed correlations with miRNA-target networks tended to be higher than those with GO and Reactome terms (see Supplementary Fig. S9). These results indicated that the miRNA-target networks associated with complex traits shared certain similarities across breeds.

**Table 1:** Between-breeds correlations based on enrichments with miRNA-target networks.

	FY	MY	PY	BC	Mastitis	Health	Fertility
HOL vs. RDC	0.45*	0.41***	0.38***	0.11***	0.23**	0.11	0.08
HOL vs. JER	0.11	0.22*	0.02	0.39***	0.17*	0.04	0.08˙
RDC vs. JER	0.12**	0.23***	0.15***	0.07˙	0.02	0.24***	0.11˙

Note: FY, MY, PY and BC are fat yield, milk yield, protein yield and body conformation, respectively. The significant levels of correlations were determined acorrding to the 1000 correlations calculated using random-target networks, assuming the correlation approximately follows a normal distribution.

***is *P* < 0.001

**is *P* < 0.01

*is *P* < 0.05, and ˙is *P* < 0.1.

### Significant miRNA target-networks

**Figure 5:**
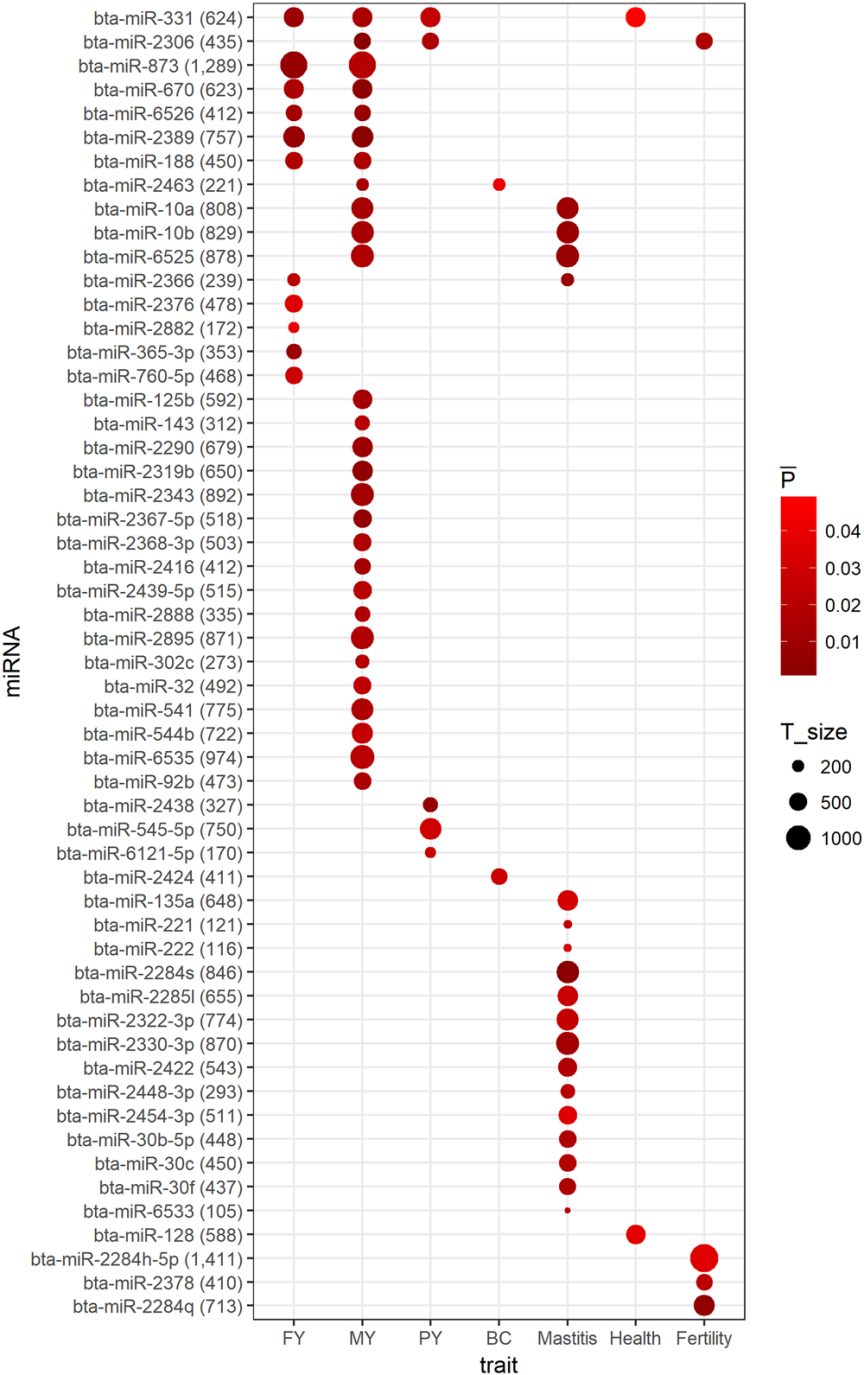
Significant miRNA-target networks for seven traits in three breeds. FY, MY, PY and BC are fat yield, milk yield, protein yield and body conformation, respectively. A miRNA target-network with *P* values < 0.05 across three breeds for the same trait was considered as significant for this particular trait. $\bar{{\rm{P}}}$ is the mean of *P* values (based on marker-set test) of a significant miRNA-target network in three breeds. T_size is the number of targets in a miRNA target-network.

**Figure 6:**
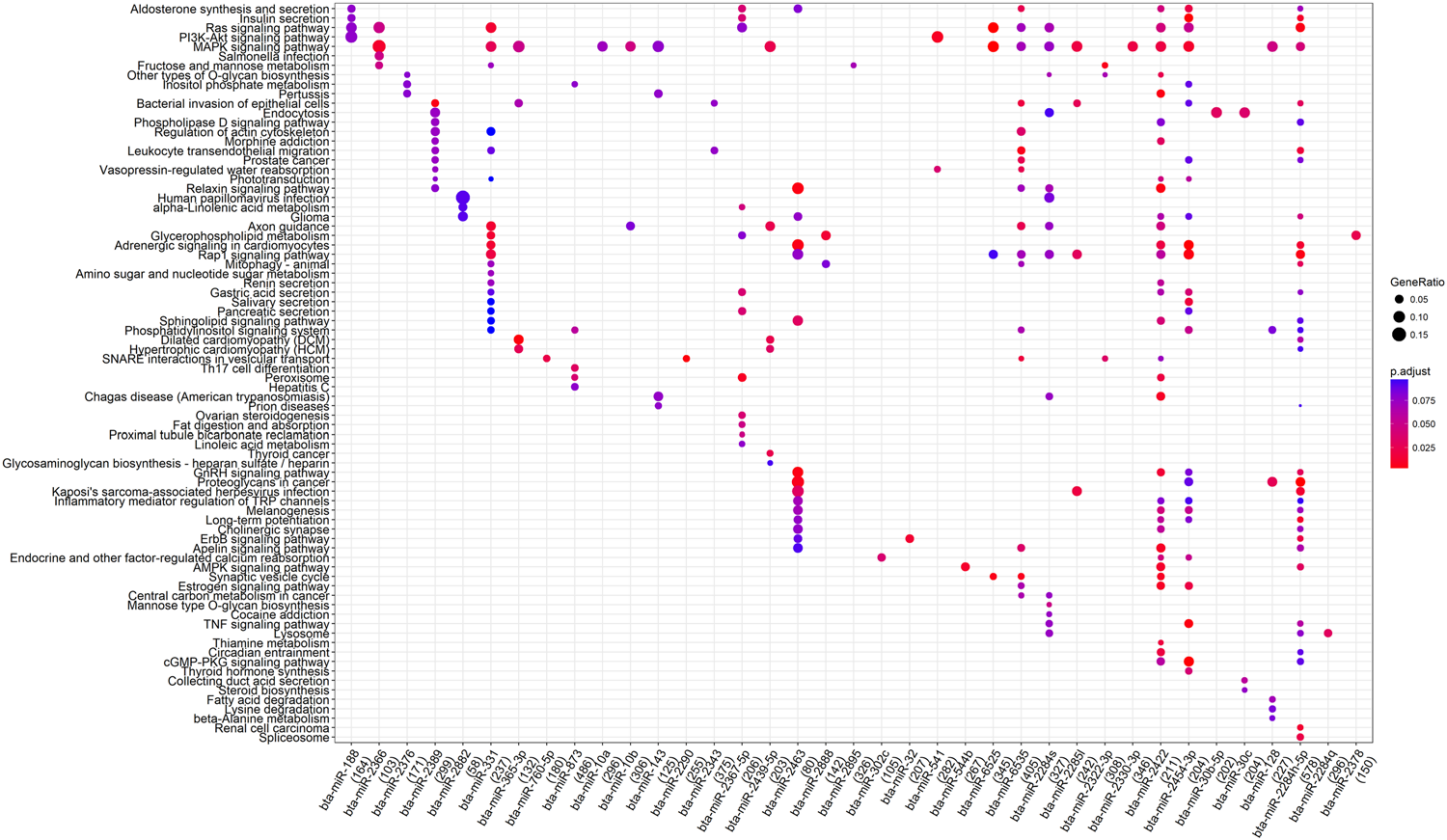
Enriched (p.adjust < 0.1) KEGG pathways for all significant miRNA-target networks. *p*.*adjust* is the corrected *P*-value using FDR methods. Values in the brackets are the number of targets that can be annotated in KEGG databases in the corresponding miRNAs, and GeneRatio is the proportion of enriched targets in a pathway over all the targets that were annotated in KEGG (values in the brackets).

When a miRNA target-network had *P*-values < 0.05 (based on marker-set test) across all three breeds for a trait (*P* < 0.05^3^ = 1.25e-04), this miRNA target-network was considered significant, suggesting that this miRNA and its target genes may participate in biological processes pertinent to this particular trait. A total of 55 significant miRNA-target networks were detected for seven traits and twelve miRNAs were involved in several traits (Fig. 5). The enriched (FDR < 0.1) KEGG pathways for the targets of these significant miRNAs are shown in Fig. 6. Thus, six miRNAs were shared by FY and MY, including bta-miR-188, bta-miR-2389, bta-miR-331, bta-miR-6526, bta-miR-670 and bta-miR-873, the targets of which were mainly engaged in MAPK signaling pathway, Ras signaling pathway, inositol phosphate and glycerophospholipid metabolism. Three miRNAs, bta-miR-10a/b and bta-miR-6525, were shared by MY and mastitis, and bta-miR-2366 was shared by FY and mastitis, the targets of which were engaged in MAPK signaling pathway, AMPK signaling pathway, Ras signaling pathway, salmonella infection, and fructose and mannose metabolism. Also, bta-miR-2306, was shared by MY, PY and fertility. Our results were in line with previous studies on miRNA transcriptomes in the mammary gland during lactating and infection^39–42^, suggesting it might be a promising way to detect candidate miRNAs for a trait through investigation of the enrichments of their targets for GWAS signals.

### Integrative analyses of infection-induced transcriptomes provide novel insights into the genetic and biological basis underlying mastitis

**Figure 7:**
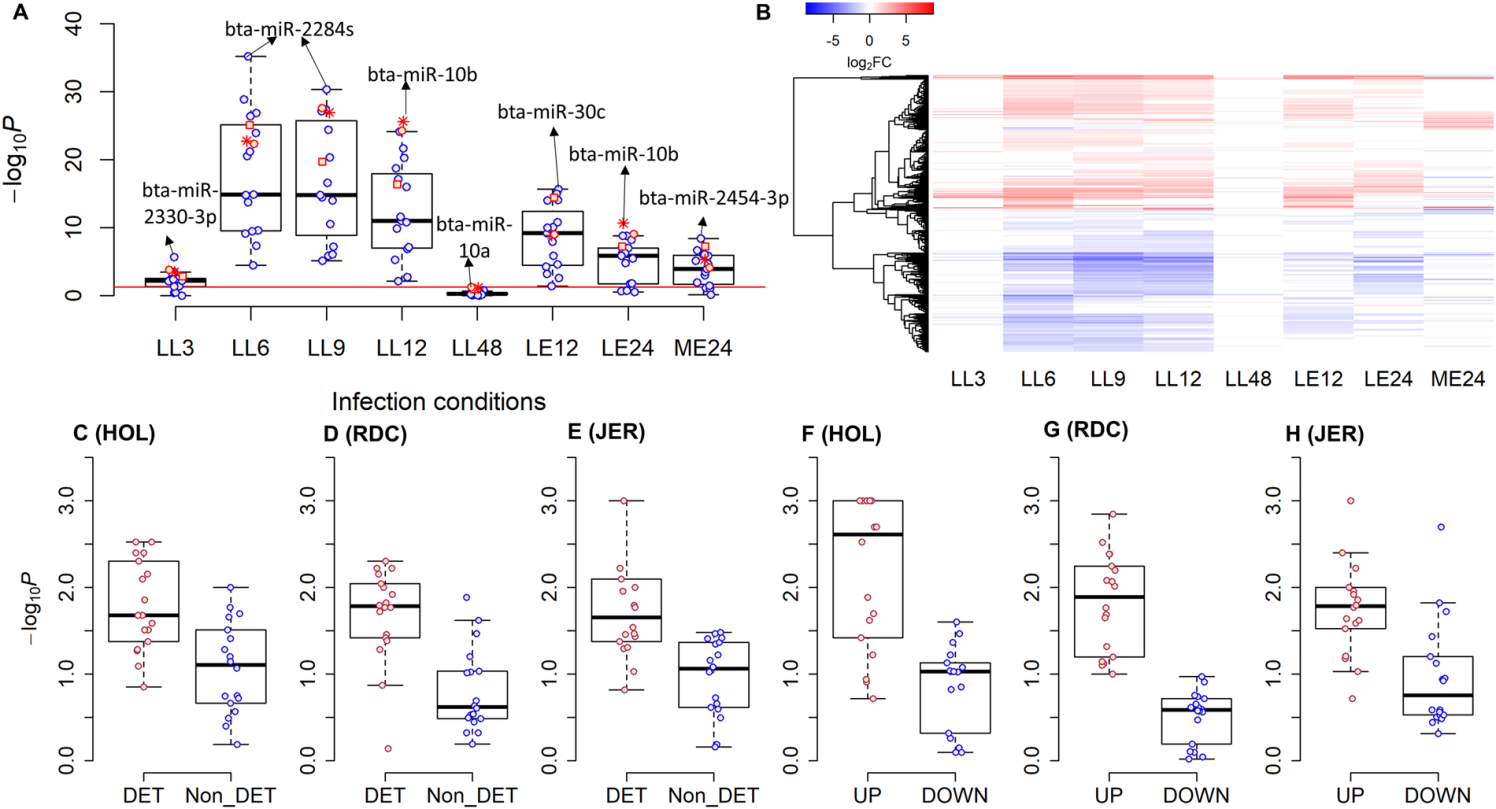
Integrative analyses of infection-relevant transcriptome data with significant mastitis miRNA target-networks. (**A**) The enrichments for targets in mastitis miRNA target-networks with differently expressed genes (DEGs) that are detected in different infection conditions. Each point is a miRNA target-network. The significance (−log_10_*P*) is determined using hypergeometric test. The red line corresponds to *P* = 0.05. The red square, star and circle represent bta-miR-6525, bta-miR-10b and bta-miR-10a, respectively, which are shared between mastitis and milk yield. LL3, LL6, LL9, LL12 and LL48 represent the comparisons of 3, 6, 9, 12 and 48 h post intra-mammary infection with LPS to the control time-point (−22h), respectively in liver. LE12 and LE24 represent the comparisons of 12 and 24 h post intra-mammary infection with *E*. *coli* to the control time-point (−144h), respectively in liver. ME24 represents the comparison of the infected udder quarters to the control ones at 24 h post intra-mammary infection with *E*. *coli*. (**B**) The heatmap of log_2_(fold-change, FC) of differentially expressed target (DETs) of mastitis-associated miRNAs. (**C**–**E**) Represent the difference in the enrichments of association signals of mastitis between DETs and non-DE targets (N_DETs) of mastitis-associated miRNAs for HOL, RDC and JER, respectively. (**F**–**H**) Represent the difference in the enrichments of association signals of mastitis between up- (UP) and down- (DOWN) regulated DETs of mastitis-associated miRNAs for HOL, RDC and JER, respectively.

We detected eighteen miRNA-target networks that were associated with mastitis (Fig. 5). To provide new insight and to further improve prioritization of GWAS signals for mastitis, we integrated these associations with liver and mammary gland transcriptome data from two intra-mammary infection experiments performed in HOL cows. Differentially expressed genes (DEGs, FDR < 0.05) in the mastitis-related transcriptomes were identified at different time-points post infeciton relative to control groups (Fig. 7, see Methods). Enrichment analysis showed that the mastitis-associated miRNA-target networks were significantly enriched (*P* < 0.05, hypergeometric test) with DEGs detected in most of the conditions (Fig. 7A), supporting that these miRNAs could be involved in the response to mastitis. The top enriched miRNA in each condition is indicated in Fig. 7A. Targets of mastitis miRNAs that were observed differentially expressed in at least two infection conditions were considered as differentially expressed targets (DETs). The heat-map of log_2_ (fold change, FC) of DETs across all experimental conditions are shown in Fig. 7B. The numbers of DETs were 2,381 unique genes and the average number of DETs per miRNAs was 326 (range: 61 to 530). The results showed consistently across all three breeds that the mastitis GWAS signals were significantly more enriched in DETs than in non-DETs (*P* < 0.05, *t*-test) (Fig. 7C–E).

**Figure 8:**
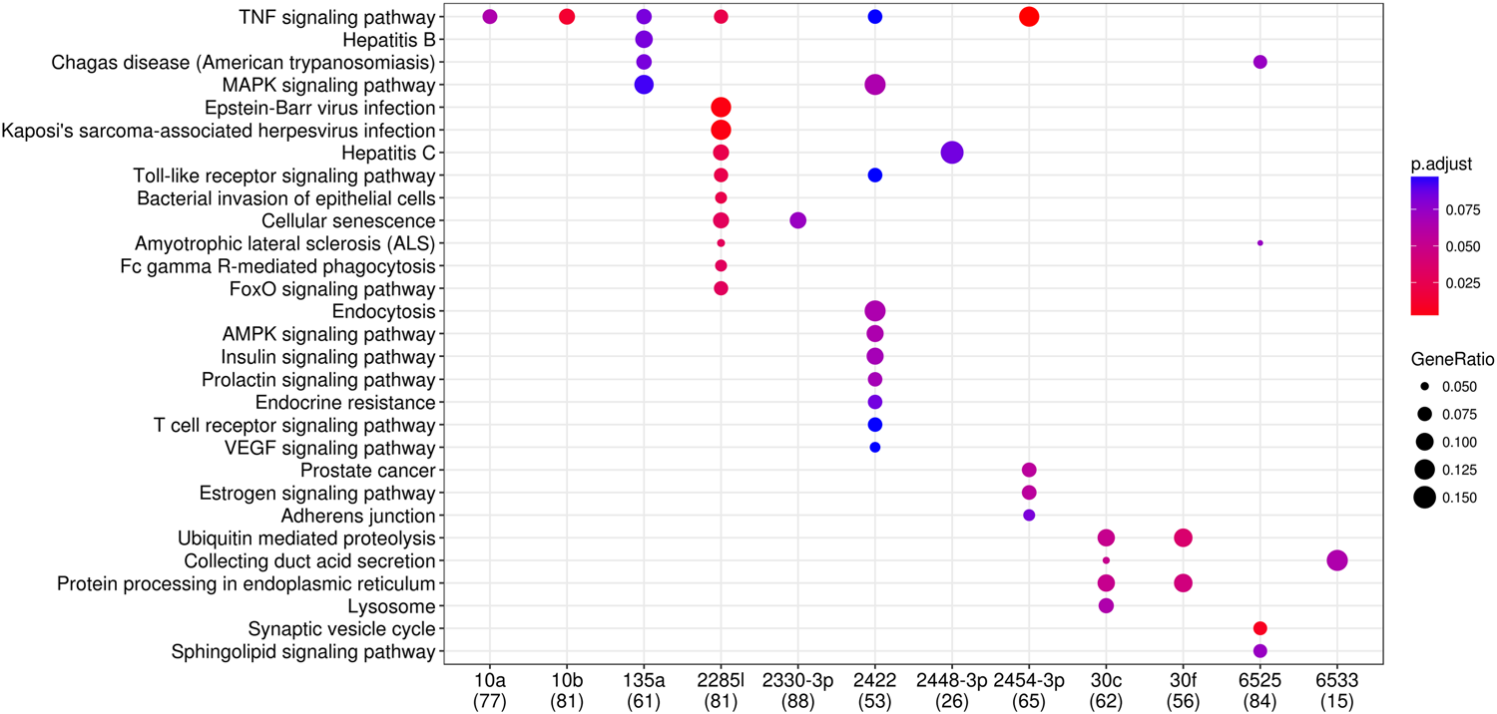
Functional enrichment analysis of up-regulated differentially expressed targets (DETs) of significant mastitis miRNAs. *p*.*adjust* is the corrected *P*-value using FDR methods. Values in the brackets are the number of up-regulated DETs that can be annotated in KEGG databases for the corresponding miRNAs, and GeneRatio is the proportion of enriched up-regulated DETs in a pathway over all the up-regulated DETs that were annotated in KEGG (values in the brackets).

Next, the DETs of these miRNAs were separated into up- and down- regulated transcripts based on their log_2_FC > 0 (up) or <0 (down) across all infection conditions. In total, 1,002 unique up-regulated DETs were detected with an average of 142 (range: 31 to 231) for each miRNA, and 1,111 down-regulated DETs were detected with an average of 146 (range: 24 to 255) for each miRNA. The mastitis GWAS signals were significantly more enriched in up-regulated DETs of miRNAs than in down-regulated DETs across all three breeds (Fig. 7F–H). In agreement, the functional enrichment analyses based on KEGG pathways demonstrated that the up-regulated DETs of these mastitis miRNAs were mainly engaged in the innate immune system, infection defense and inflammatory response (Fig. 8).

### Bta-miR-10a/b and bta-miR-6525 are associated with MY and mastitis

**Figure 9:**
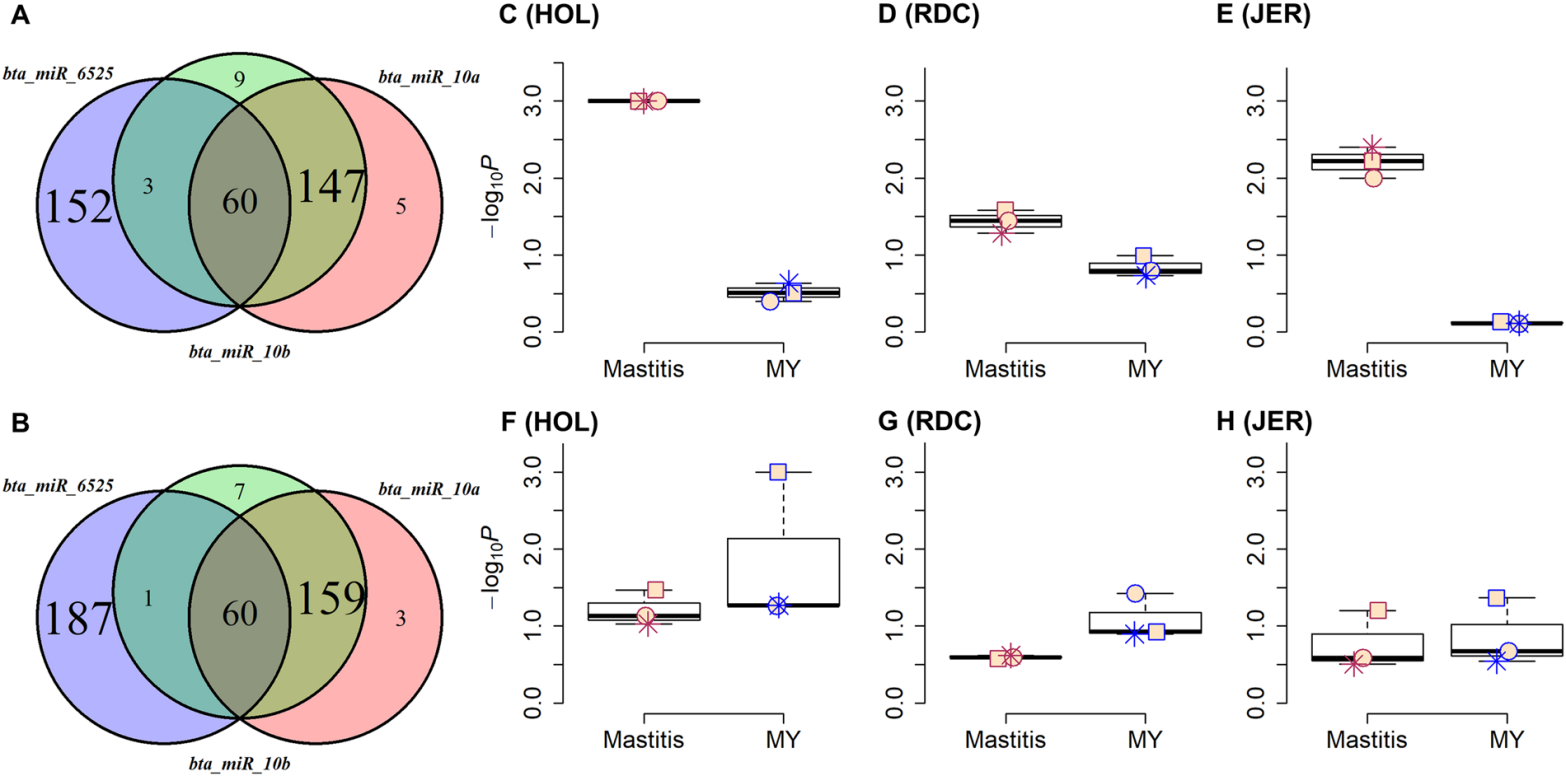
Up- and down-regulated differentially expressed targets (DETs) of bta-miR-10a/b and bta-miR-6525. (**A**,**B**) are overlaps of up-/down- regulated DETs of bta-miR-10a/b and bta-miR-6525 respectively. (**C**–**E**) Are the difference in their up-regulated DETs in enrichments (−log_10_*P*) of association signals between mastitis and milk yield (MY) for HOL, RDC and JER, respectively, while (**F**–**H**) are the difference in the down-regulated DETs in enrichments of association signals between mastitis and MY for HOL, RDC, and JER, respectively. The red square, star and circle represent bta-miR-6525, bta-miR-10b and bta-miR-10a, respectively.

**Figure 10:**
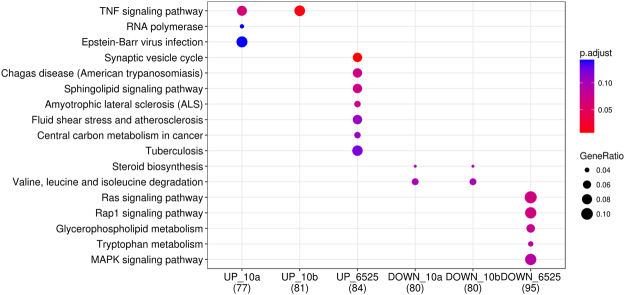
Functional enrichment analyses of up-/down-regulated differentially expressed targets (DETs) of bta-miR-10a/b and bta-miR-6525. *p*.*adjust* is the corrected *P*-value using FDR methods. Values in the brackets are the number of DETs that can be annotated in KEGG databases, and GeneRatio is the proportion of enriched DETs in a pathway over all DETs that were annotated in KEGG (values in the brackets). UP_10a, UP_10b and UP_6525 correspond to up-regulated DETs of bta-miR-10a, bta-miR-10b, bta-miR-6525, respectively, while DOWN_10a, DOWN_10b and DOWN_6525 correspond to down-regulated DETs of bta-miR-10a, bta-miR-10b, bta-miR-6525, respectively.

Three miRNA target networks, bta-miR-10a, bta-miR-10b and bta-miR-6525, were enriched for GWAS signals for both MY and mastitis. Integration with mastitis-related transcriptome data showed that transcripts that are up-regulated for all three miRNAs (Fig. 9A) were significantly more enriched with association signals for mastitis than for MY across the three breeds (Fig. 10C–E), while the down-regulated DETs (Fig. 9B) tended to be more associated with MY than with mastitis across all breeds (Fig. 9F–H). Consistently, functional analyses of up- and down-regulated DETs demonstrated that the up-regulated DETs of the three miRNAs were mainly engaged in inflammation and immune responses such as TNF signaling pathway and Epstein-Barr virus infection, while their down-regulated DETs were mainly engaged in amino acid metabolism, such as steroid biosynthesis and valine, leucine and isoleucine degradation (Fig. 10). These results were in agreement with previous reports showing involvement of bta-miR-10a and bta-miR-10b not only in innate immune responses but also in lipid and cholesterol metabolism^43–49^.

## Discussion

### Knowledge-guided genotype-phenotype mapping and genomic prediction

Identifying and utilizing causal links between genotypes and quantitative phenotypes and diseases is a major goal in animal breeding as well as in clinical personalized medicine. Multiple statistical models have been used for genotype-phenotype mapping, and the performance of the models depends critically on the genetic architecture underlying complex traits. Single-marker regression models are suitable for detection of genomic variants with large effects but not for identifying variants with small effects^50^, and therefore typically accounts for only a fraction of the total genetic variance. Knowledge-guided gene-mapping approaches such as marker-set test based on single-marker statistics or genomic feature prediction models such as GFBLUP^9^ and BayesRC^51^ can contribute to the discovery of the so-called “missing heritability” in the standard GWAS. The sum-based marker-set test method applied in this study has been demonstrated to have higher power or at least equal to most commonly used marker-set test methods, particularly for the highly polygenic traits^16,18^. Provided the genomic features are enriched for causal variants, incorporating this type of biological information into the genomic prediction model can also improve the prediction accuracy^9,17,18^. Thus, previous studies demonstrated a positive correlation between association signal enrichment and the predictive ability of GFBLUP model that allowed us to differentially weight SNPs associated with that particular genomic feature^11,18^. In fact, any biological information that can be linked to specific genomic regions containing, or in LD with, SNPs, can be used to construct the genomic features. Important sources for defining trait-relevant features include the genome, epigenome, transcriptome, proteome and metabolome levels, or functional networks such as KEGG pathways, gene ontology terms and protein-protein interaction complexes. Information from several resources may be utilized simultaneously to capture the dynamic aspects of genome function. Clearly with the increasing availability of large-scale GWAS results and more comprehensive genome annotation, the marker-set test and the genomic feature based prediction models will be increasingly useful in the near future for identifying associations between a trait and a genomic feature and for improving genomic prediction of complex phenotypes.

### MicroRNAs and their target networks contribute to genetic variations in complex traits

MicroRNAs are functional noncoding RNAs that modulate translation and decay of mRNA transcripts, thereby playing major roles as regulators of gene expression in a wide range of biological processes. Here we interrogated data from genome-wide association studies for evidence of overrepresentation of miRNA genes and their target genes. Our analysis revealed significant enrichment of GWAS signals in proximity to miRNA genes for complex traits in all three dairy cattle breeds. Overall, 55 significant miRNA-target networks were detected for the seven traits, out of which 12 were shared among multiple traits, indicating pleiotropic effects of miRNAs (Fig. 5). Our observations agree well with a number of other studies that addressed the roles of miRNAs in mammary gland development and lactation. Thus, a recent analysis of miRNA expression in milk during the entire lactation cycle detected a total 475 known and 348 novel miRNAs. Comparison between different cycle stages revealed 344, 366, and 209 differentially up- and down-regulated expression of miRNAs between lactogenesis and galactopoiesis, involution and galactopoiesis, and involution and lactogenesis, respectively^52^. Likewise, expression profiling of lactating versus non-lactating stages of mammary glands identified a total of 900 known and candidate microRNAs of which more than 60% were shared between the two periods^53^. The high diversity and abundance, as well as the differentially regulated expression patterns of miRNAs in mammary gland tissue clearly underscores that synthesis and secretion of milk as well as switches between lactation stage involves a high level of posttranscriptional regulation of gene expression by miRNAs.

Eighteen miRNA-target networks were associated with mastitis many of which have previously been observed in connection with mammary gland infections. Thus, *Streptococcus agalactiae*-induced mastitis of bovine mammary glands resulted in altered expression of thirty five miRNAs including a bta-miR-30 family member, bta-miR-135a, and bta-miR-2284 family members^41^. Challenge of bovine MAC-T mammary epithelial cells with pathogenic *Staphylococcus aureus* or *Escherichia coli* led to differential expression of seventeen miRNA genes among which were bta-miR-30b-5p and bta-miR-30c^39^. A total of 77 miRNAs that included bta-miR-2422, bta-miR-135a, a bta-miR-2285 member, and several bta-miR-2284 family members showed expression differences in *Staphylococcus aureus* infected mammary glands compared to control groups^42^. Furthermore, miRNA expression profiles of milk exosomes from cows prior and after infection of the mammary gland with *Staphylococcus aureus* showed significant changes in fourteen known genes including bta-miR-10a^40^. Also bta-miR-222 expression was significantly upregulated in mastitis-affected cows^54^. Co-expression network and pathway analysis revealed significant correlations between milk somatic cell count (SCC), an indicator trait for mastitis, and bta-miR-10a, bta-miR-2448, bta-miR-2284 family members and bta-miR-2285 family members^55^. Also in other species several of these eighteen miRNAs have been linked to mastitis and immune protection. In porcine mammary epithelial cells, coliform mastitis induced differential expression of miR-10a/10b and miR-30b/c/f^56^. Moreover, miR-221 was differentially expressed in colostrum and peak lactation periods in the caprine mammary gland^57^.

Finally we defined genomic features for mastitis by integrating both transcriptome and miRNA-target networks with GWAS data. The results showed consistently across all three breeds that the mastitis associated GWAS signals were significantly more enriched in DETs than in non-DETs. Additionally, partitioning of DETs showed that up-regulated targets had higher enrichment compared to down-regulated targets. Furthermore, three miRNA-target networks showed enrichment for GWAS signals for both MY and mastitis. Intriguingly, when integrated with the mastitis-related transcriptome data, the analysis revealed that up-regulated transcripts belonging the network targets of all three miRNAs were significantly more enriched with association signals for mastitis than for MY across the three breeds; and vice versa, the down-regulated transcripts were more associated with MY than with mastitis. High milk yield is often associated with risk of mastitis^58^. Our work showed a genetic link between the two traits mediated by miRNA-target networks, which may in part help understanding the unfavorable correlation between mastitis and MY.

## Conclusion

In conclusion, the marker-set test of joint effects of SNPs linked to miRNAs enabled us to discover contributions from miRNAs as well as of their target networks to multiple complex traits in three cattle breeds. Overall, this work underscores that the use of biological priors such as genomic features defined by miRNAs and their targets enhances our insight into the genetic architecture underlying phenotypic diversity. With the rapid progress in the field of annotation of functional elements in livestock genomes, the genomic feature based analysis like marker-set test or GFBLUP will be increasingly useful for discovering small effect loci or for increasing the accuracy and reliability of prediction of consequences of genomic selection in animal and plant breeding.

## Materials and Methods

### Infection-induced transcriptome data

All experimental procedures involving animals have been approved by the Danish Animal Experiments Inspectorate and complied with Danish Ministry of Justice Laws concerning animal experimentation and care of experimental animals. All the experiments were performed in strict accordance with guidelines and regulations established by these committees. Members from these committees carried out inspections during the entire animal infection experiments.

All the transcriptome data used in this study came from two intra-mammary infection studies involving three and six healthy HOL animals, respectively at the very early stage of their first lactation. The details of the involved animals and the sample collection procedures were previously described by Jiang *et al*.^59^ and Jorgensen *et al*.^60^. Briefly, in the first study liver biopsies were sampled at −22, 3, 6, 9, 12, and 48 h relative to LPS intra-mammary treatment in all three studied animals (*i*.*e*., each group with three biological replicates). In the second study liver biopsies were collected from the six animals at −144, 12 and 24 h post intra-mammary infection with *E*. *coli*, and the mammary gland samples of the infected and control udder quarters were collected from the same animals at 24 h post infection (*i*.*e*., each group with six biological replicates). Finally, a total of 48 samples were applied with RNA sequencing using a 100 bp pair-end approach with llumina HiSeq 2000 by AROS Applied Biotechnology (Aarhus, Denmark). Time was considered as the only effect in the statistical model for analyzing liver samples data, while infection status was included in the model for mammary gland samples. The details of RNA-seq data analyses were previously described by Fang *et al*.^22^. FDR method was employed to control multiple testing, and genes with FDR < 0.05 were considered as DEGs.

### Phenotypes and imputed sequence genotypes

The phenotypes currently analysed were de-regressed proofs (DRPs) of seven complex traits of economic importance in cattle, including three milk production traits (*i*.*e*., MY, FY and PY), BC, mastitis, health and fertility. These DRPs were obtained from a routine genetic evaluation by Nordic Cattle Genetic Evaluation (http://www.nordicebv.info/) and were available for 5,056 HOL, 4,310 RDC and 1,231 JER bulls. All known systematic effects have been corrected.

The whole genome sequence genotypes of these animals were obtained from the imputation of 50 K and High Density (HD) genotypes. The imputation was conducted using a two-step strategy as recommended by Brøndum *et al*.^61^, and the details has been previously described by Wu *et al*.^62^. Briefly, in the first step a 50 K was imputed into a HD genotype for each individual using IMPUTE2 v2.3.1^63^ based on 3,383 reference animals (including 1,222 HOL, 1,326 RDC and 835 JER) that had been genotyped by Illumina BovineHD chips (Illumina, Inc., San Diego, CA). In the next step the imputed HD genotypes were imputed to the whole genome sequence level using *Minimac2*^64^ based on the reference population of 1,228 animals from both *Run4* of the 1,000 Bull Genomes Project^65^ and in-house whole genome sequence data^62^. In total, 22,751,039 biallelic variants were obtained after the imputation. The imputed sequence genotypes were then filtered to keep markers with deviation from Hardy-Weinberg proportions (HWP) > 10^−6^ and minor allele frequency (MAF) > 0.01. After the data editing, 15,355,382, 15,243,827 and 13,403,916 SNPs remained for the following analyses in HOL, RDC and JER, respectively with an average imputation accuracy of larger than 0.90. The imputation accuracy of a SNP increased with the increase of its MAF, and the details of the imputation accuracy were described by Wu *et al*. and Iso-Touru *et al*.^62,66^.

### Single-marker GWAS using imputed sequence data

The single-marker GWAS for the imputed sequence genotypes was conducted using a two-step variance component-based method that was implemented in EMMAX^67^. The EMMAX was developed to take account for genetic relatedness and population structure as well as to improve the computational speed. In the first step, the polygenic and residual variances were estimated by

1 $${\boldsymbol{y}}={\bf{1}}\mu +{\bf{Z}}{\boldsymbol{a}}+{\boldsymbol{e}},$$

where ${\boldsymbol{y}}$ is a vector of the phenotype (*i*.*e*., DRP); $\mu$ is the overall mean; ${\boldsymbol{a}}$ is a vector of random polygenic effects, where ${\boldsymbol{a}}$ ~ N(**0**, ${\bf{G}}{\sigma }_{a}^{2}$), and ${\bf{G}}$ is the genomic relationship matrix built using HD genotypes through excluding the chromosome containing the candidate SNP to control double fitting^68^, and ${\sigma }_{a}^{2}$ is the additive genetic variance; ${\boldsymbol{e}}$ is the vector of random residuals, where ${\boldsymbol{e}}$ ~ N(**0**,$\,{\bf{I}}{\sigma }_{e}^{2}$), and ${\bf{I}}$ is the identity matrix, and ${\sigma }_{e}^{2}$ is the residual variance. **1** is a vector of ones. **Z** is a design matrix relating phenotypes to polygenic effects. In the second step, the individual association signal was assessed by a linear regression model

2 $${\boldsymbol{y}}={\bf{1}}{\boldsymbol{\mu }}+{\boldsymbol{xb}}+{\boldsymbol{\eta }},$$

where ***y***, ***μ*** and **1** are the same as described above, ${\boldsymbol{x}}$ is a vector of genotype dosages (range: from 0 to 2), ${\boldsymbol{b}}$ is the allele substitution effect, and ${\boldsymbol{\eta }}$ is a vector of random residual deviates with (co)variance structure ${\bf{G}}{\sigma }_{a}^{2}$+$\,{\bf{I}}{\sigma }_{e}^{2}$. The genome-wide significance thresholds corresponding to an error rate of 0.05 (Bonferroni correction) were set at 3.3 × 10^−9^, 3.3 × 10^−9^, 3.7 × 10^−9^ for HOL, RDC and JER, respectively. Manhattan plots were generated using *qqman* v.0.1.2 implemented in the R package^69^.

Regions showing association signal could be very large due to high LD structure owing to small effective population sizes in dairy cattle breeds. Therefore, we demarcated a region around the top SNP, which most likely harbours the causal mutation. A QTL region boundaries were defined through extending the position of the top SNP up- and down- stream to include SNPs with −log10(*P*-values) higher than −log10(*P*-value) minus three of the top SNP^70^. The proportion of genomic variance explained by the top SNP within a QTL was calculated as $2pq{b}^{2}/{\sigma }_{a}^{2}$, where $p$ and $q$ were allele frequencies, *b* was the allele substitution effect, and ${\sigma }_{a}^{2}$ was the additive genomic variance.

### Marker sets defined from GO and Reactome databases

Genes grouped into a specific GO or Reactome term were considered to be a single marker set (*i*.*e*., genomic feature). The Bioconductor package “org.Bt.eg.db” v. 3.3.0 (https://bioconductor.org/packages/release/data/annotation/html/org.Bt.eg.db.html) and “reactome.db” v 3.3.0 (https://bioconductor.org/packages/release/data/annotation/html/reactome.db.html) were used to link genes to the GO and Reactome terms, respectively. Here, we focused on the GO and Reactome terms consisting of at least 10 directly evidenced genes. In GO terms, only biological processes terms were considered. The imputed sequence variants were mapped to the bovine reference genome (UMD3.1). A SNP was assigned to a gene if its chromosome position was between the start and end positions of the gene (*i*.*e*., within the open reading frames, ORF).

### Marker-set test

Since the genomic variance of the studied complex traits has been typically considered to be controlled by many loci of small to moderate effects, the following sum-based summary statistics for a genomic feature was chosen, and it has been demonstrated to have higher power or at least equal to most commonly used marker-set test methods, particularly for the highly polygenic traits^16,18,21^.

3 $${T}_{sum}=\sum _{i=1}^{{m}_{f}}{t}^{2},$$

In which ${{\rm{m}}}_{{\rm{f}}}$ is the number of SNPs in a genomic feature, and ${t}^{2}$ is the square of *t* that was calculated as the SNP effect (*b*) divided by its standard error. It should be noticed that the LD patterns of SNPs among multiple genes in a genomic feature were typically low, especially for genes in different chromosomes. Although the LD patterns among SNPs and the sizes of marker sets were not directly taken into account in the summary statistics, they have been taken in account by the following cyclical permutation strategy as previously described^21^. Briefly, the test statistics (*i*.*e*., ${t}^{2}$) for all SNPs were ordered according to their chromosome positions (*i*.*e*., ${t}_{1}^{2}$, ${t}_{2}^{2}$, $\cdots$ ${t}_{m-1}^{2}$, ${t}_{m}^{2}$,). A random test statistic (*i*.*e*., ${t}_{k}^{2}$,) was chosen from this vector as the first, and the remaining test statistics were shifted to new positions, but retained their original orders (*i*.*e*., *t*_k_, *t*_k+1_ $\cdots$ *t*_*m*_, *t*_1_ $\cdots$ *t*_k−1_). Therefore, any association of SNPs with genomic features was uncoupled while maintaining the correlation structure among test statistics of SNPs. Afterwards, a new summary statistic of the genomic feature was computed according to its original chromosome position. The permutation procedure was repeated 1,000 times for each tested genomic feature, and an empirical *P*-value was computed according to one-tailed tests of the proportion of random summary statistics larger than that observed. This marker set-test method together with multiple quantitative genomic tools have been implemented in our QGG package (http://psoerensen.github.io/qgg/).

### Significant testing for between-traits/breeds correlations using random-target networks

The between-trait correlations within a breed and the between-breed correlations for a same trait were computed based on the GWAS signal enrichments of miRNA target-networks. In order to determine the significant level of an observed correlation, we randomly generated targets with the same size as predicted for each miRNA, then a random correlation was calculated using the enrichments of random target-networks. We repeated this procedure for 1000 times, thereby 1000 random correlations were generated for each particular correlation. Assuming that a correlation approximately follows a normal distribution *r* ~ *N*(µ, σ^2^), where µ and σ^2^ can be approximately estimated as the mean and standard deviation (SD) of the 1000 randomly generated correlations. The significant level for an observed correlation based on miRNA target-networks was then determined by using one-sided test.

### Functional enrichment analyses of gene lists

The functional enrichment analyses of a list of genes were conducted using R package clusterProfiler^71^, where a hypergeometric test, based on a KEGG pathway database, was applied, and the *P*-values for each pathway were adjusted using the FDR method. We used pathways with FDR < 0.1 to indicate the putative biological function of gene lists being studied.

### Availability of data and materials

All genomic annotation data defining gene regions is available for download (ftp://ftp.ensembl.org/pub/release-84/gtf/bos_taurus). The miRNA annotation data is available for download (http://www.mirbase.org/ftp.shtml), and their best quantile predicted target genes is available in (http://mirmap.ezlab.org/). The GO annotation database can be publicly accessed (https://bioconductor.org/packages/release/data/annotation/html/org.Bt.eg.db.html). The Reactome annotation database can be publicly accessed (http://bioconductor.org/packages/release/data/annotation/html/reactome.db.html). The whole genome sequencing data from the 1000 Bull Genomes Project are publicly available from NBCI under SRA no. SRP039339 (http://www.ncbi.nlm.nih.gov/bioproject/PRJNA238491) and variations in dbSNP (http://www.ncbi.nlm.nih.gov/projects/SNP/). The genotype and phenotype data, and genome sequence data from Aarhus University are available only upon agreement with the commercial breeding organization (http://www.vikinggenetics.com/) and should be requested directly from the authors or the breeding organization.

## Electronic supplementary material

Supplementary infomation
