# Supplementary material for: MicroRNA-guided prioritization of genome-wide association signals reveals the importance of microRNA-target gene networks for complex traits in cattle
Source: Sci Rep. 2018 Jun 19;8:9345. doi: 10.1038/s41598-018-27729-y (PMC6008395; doi:10.1038/s41598-018-27729-y)
Supplement: Supplementary file 1 — Supplementary infomation [file 41598_2018_27729_MOESM1_ESM.pdf]

MicroRNA-guided prioritization of genome-wide association signals reveals the importance of microRNA-target gene networks for complex traits in cattle

Lingzhao Fang[1,3,4,5*], Peter Sørensen[1], Goutam Sahana[1], Frank Panitz[2], Guosheng Su[1], Shengli Zhang[3], Ying Yu[3], Bingjie Li[1], Li Ma[4], George Liu[5], Mogens Sandø Lund[1], Bo Thomsen[2]

[1]Center for Quantitative Genetics and Genomics, Department of Molecular Biology and Genetics, Aarhus University, 8830 Tjele, Denmark

[2]Section for Molecular Genetics and Systems Biology, Department of Molecular Biology and Genetics, Aarhus University, 8000 Aarhus C, Denmark

[3]Key Laboratory of Animal Genetics, Breeding and Reproduction, Ministry of Agriculture & National Engineering Laboratory for Animal Breeding, College of Animal Science and Technology, China Agricultural University, 100193, Beijing, China

[4]Department of Animal and Avian Sciences, University of Maryland, College Park, 20742 MD, USA

[5]Animal Genomics and Improvement Laboratory, ARS USDA, Beltsville, Maryland, USA

[*]**Corresponding Author:** lzfang@umd.edu

Peter Sørensen: pso@mbg.au.dk

Goutam Sahana: goutam.sahana@mbg.au.dk

Frank Panitz: frank.panitz@mbg.au.dk

Guosheng Su: guosheng.su@mbg.au.dk

Shengli Zhang: zhangslcau@cau.edu.cn

Ying Yu: yuying@cau.edu.cn

Bingjie Li: Bingjie.li@mbg.au.dk

Li Ma: lima@umd.edu

George Liu: George.Liu@ars.usda.gov

Mogens Sandø Lund: mogens.lund@mbg.au.dk

Bo Thomsen: bo.thomsen@mbg.au.dk

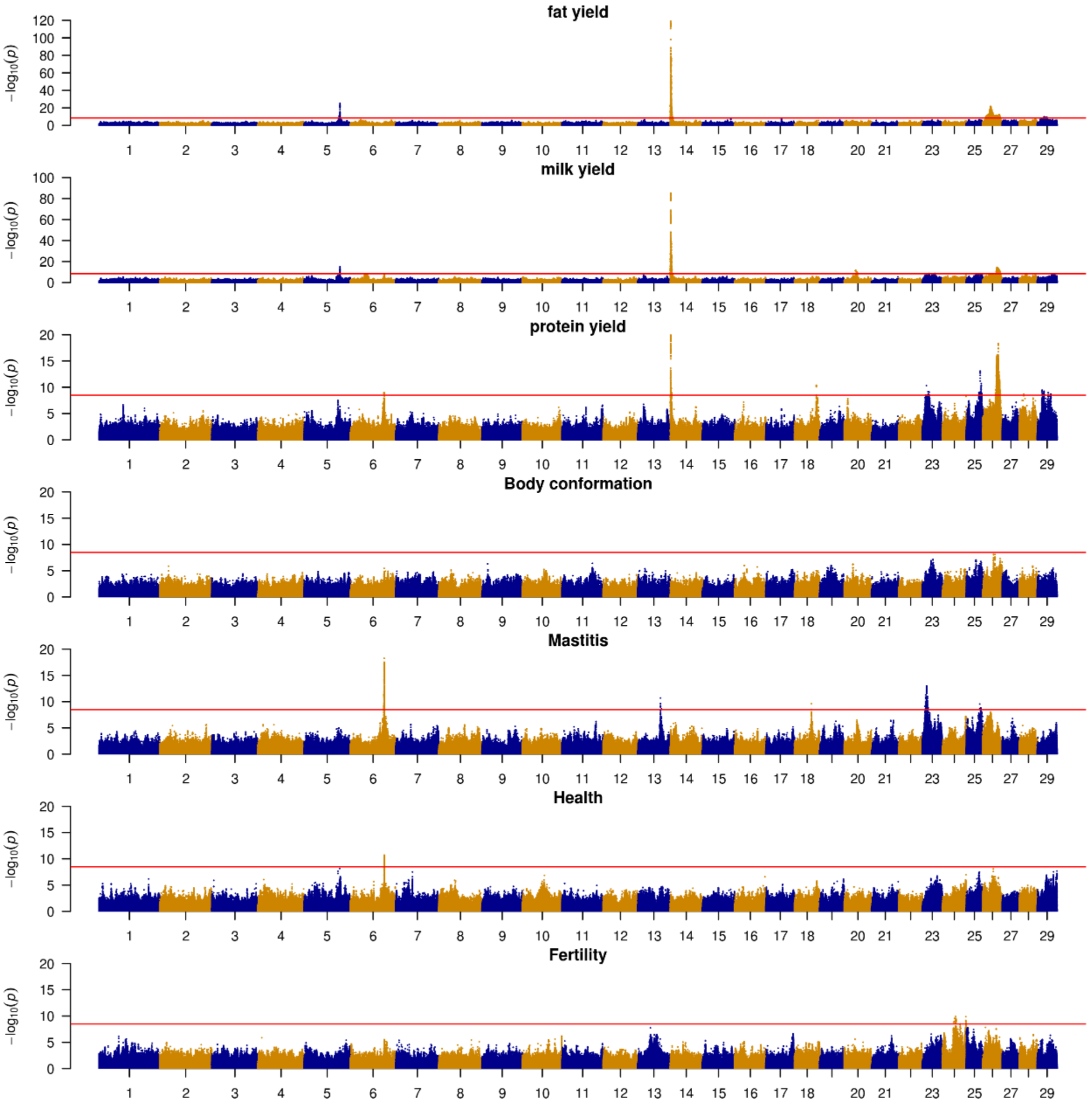

Supplementary Fig. S1. Manhattan plot for sequence-based genome-wide association study (GWAS) of seven traits in Holstein (HOL)

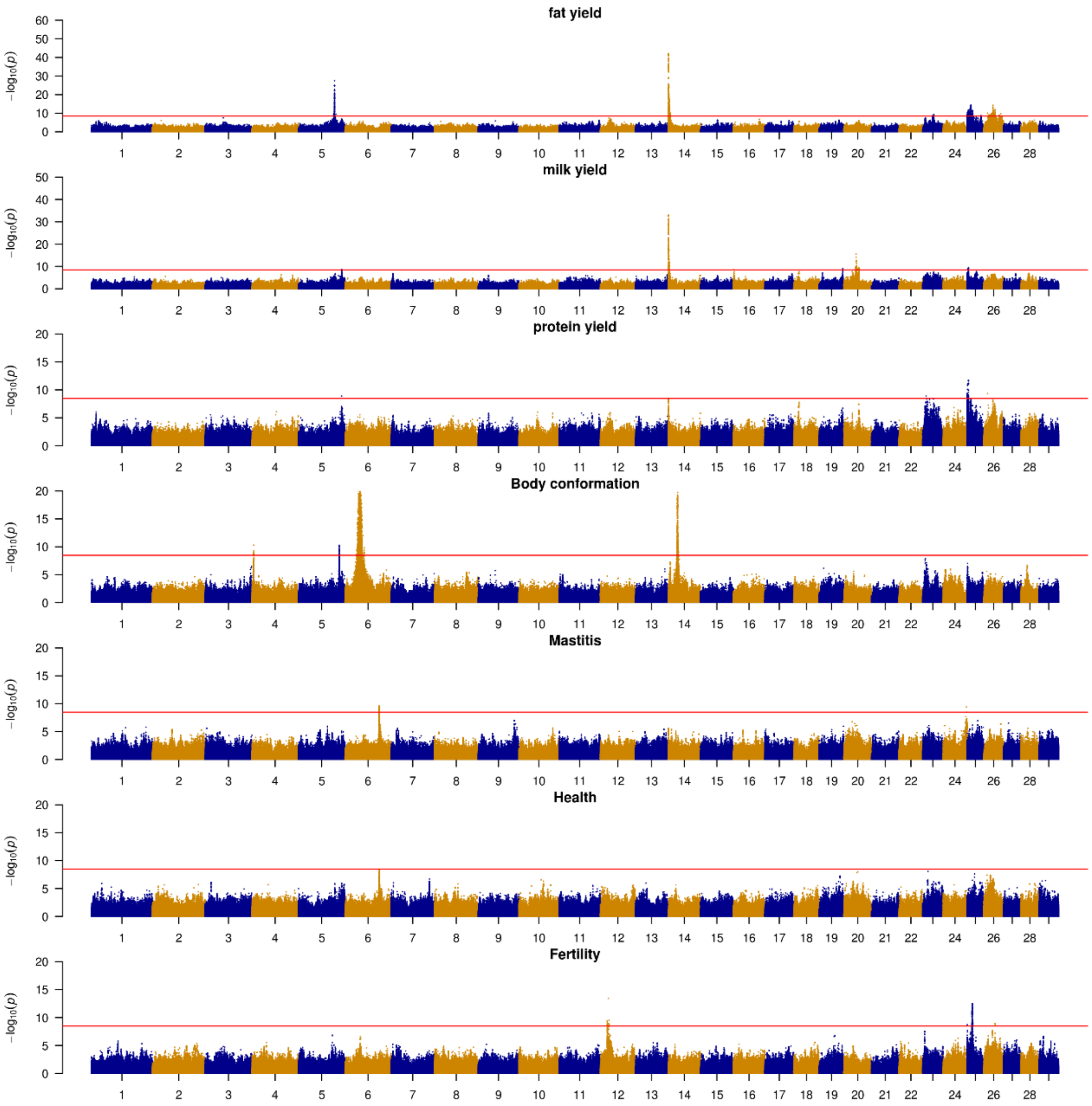

Supplementary Fig. S2. Manhattan plot for sequence-based genome-wide association study (GWAS) of seven traits in Nordic Red cattle (RDC)

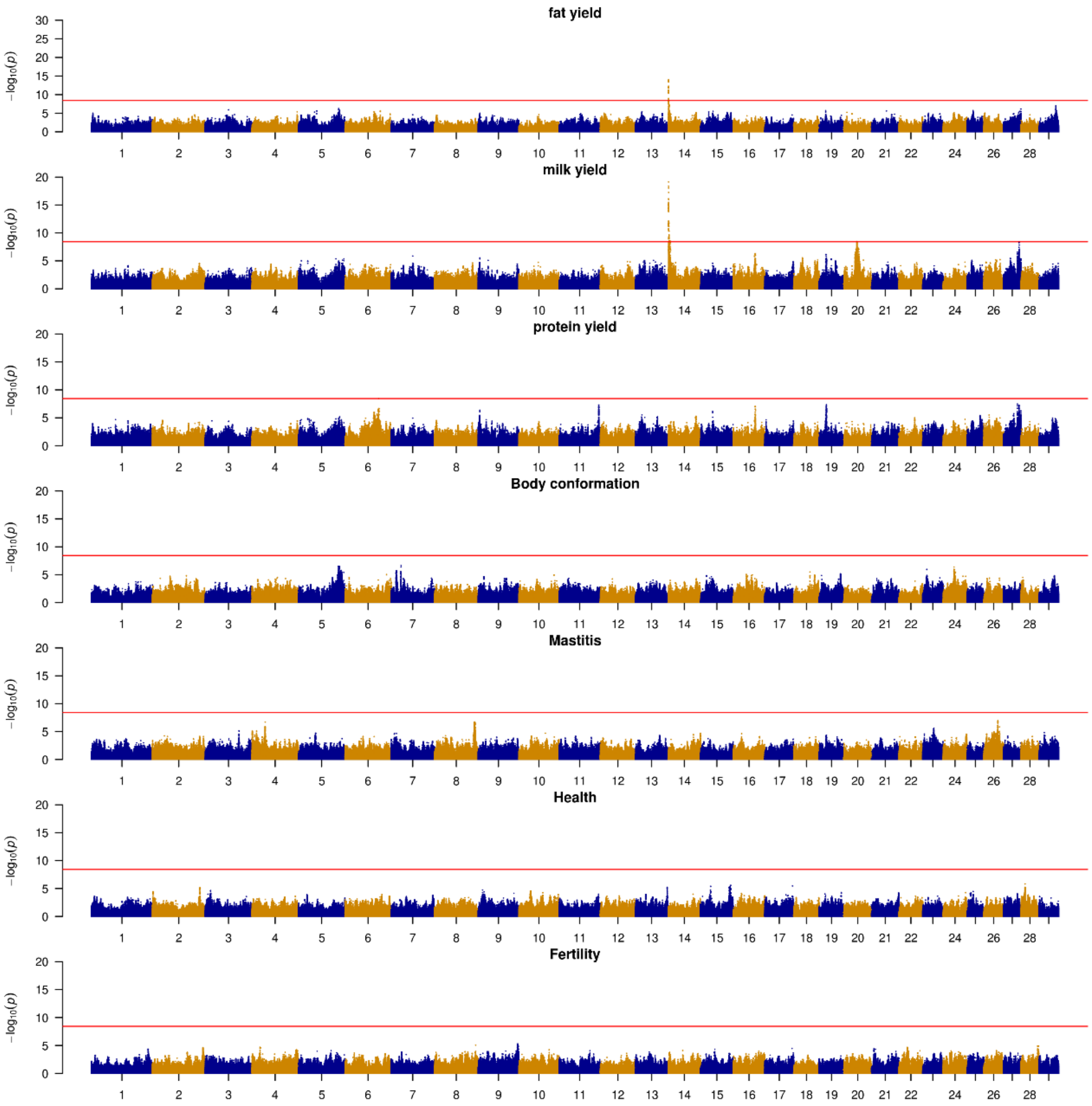

Supplementary Fig. S3. Manhattan plot for sequence-based genome-wide association study (GWAS) of seven traits in Jersey (JER)

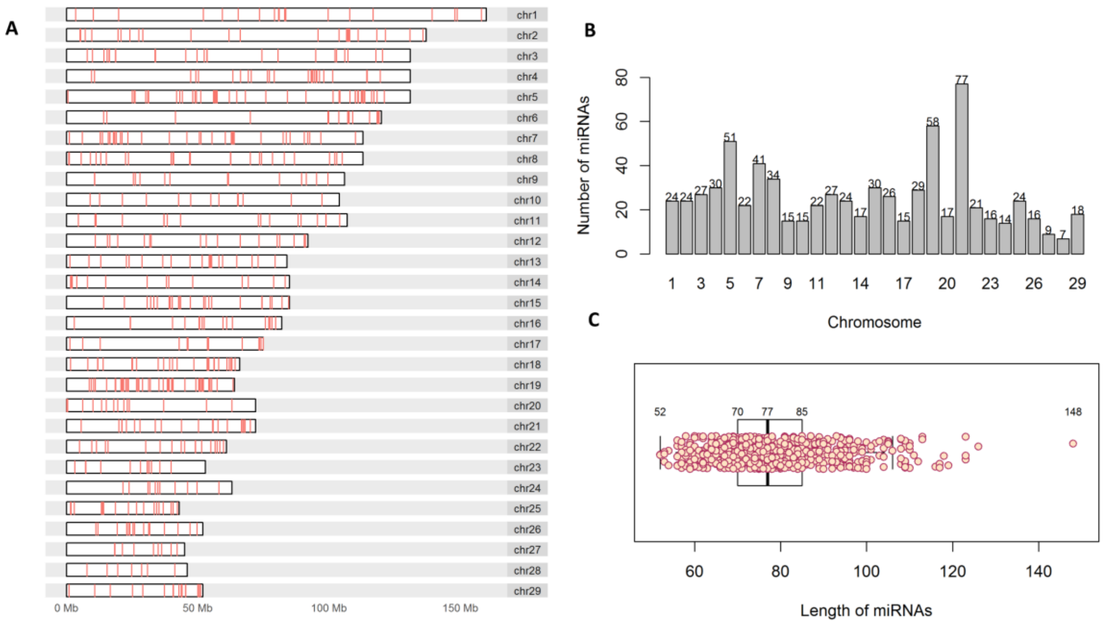

Supplementary Fig. S4. General characteristics of 750 bovine autosome miRNA genes. A is the distribution of miRNA genes along the bovine genome, and each red bar is a miRNA gene. **B** is the number of miRNA genes on each chromosome. **C** is the length (base-pair, bp) of miRNA genes, and each dot is a miRNA gene.

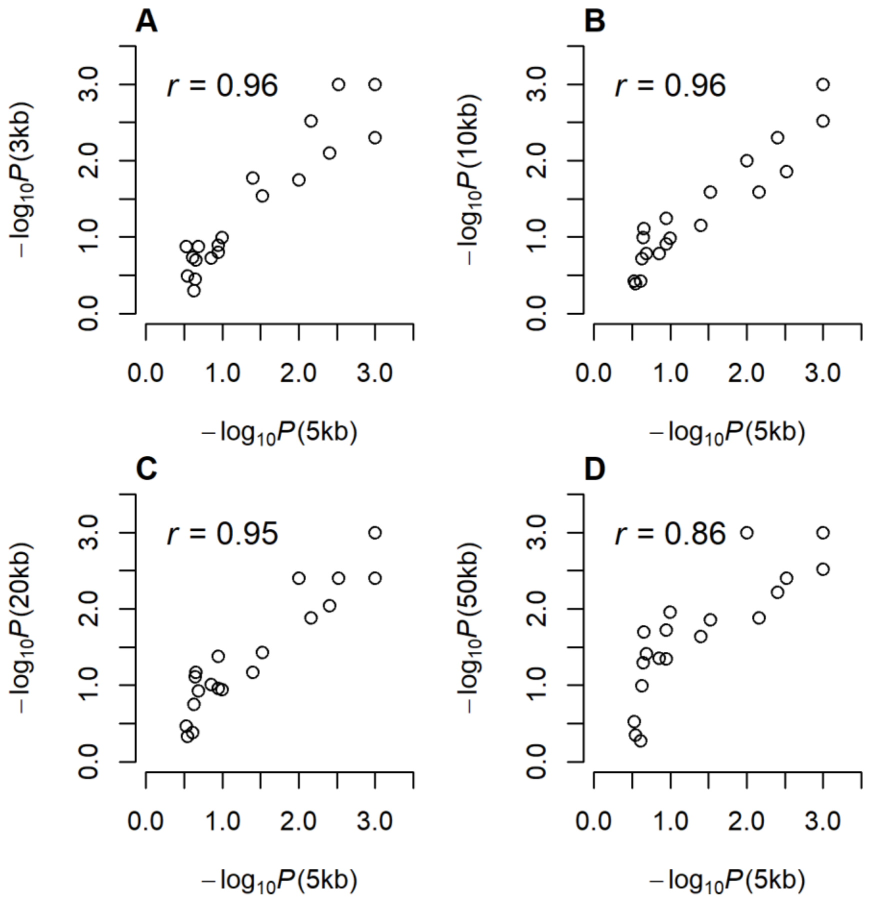

Supplementary Fig. S5. Correlations of –$\log_{10}$ *P* values (marker-set test) between different extensions in seven traits among three breeds

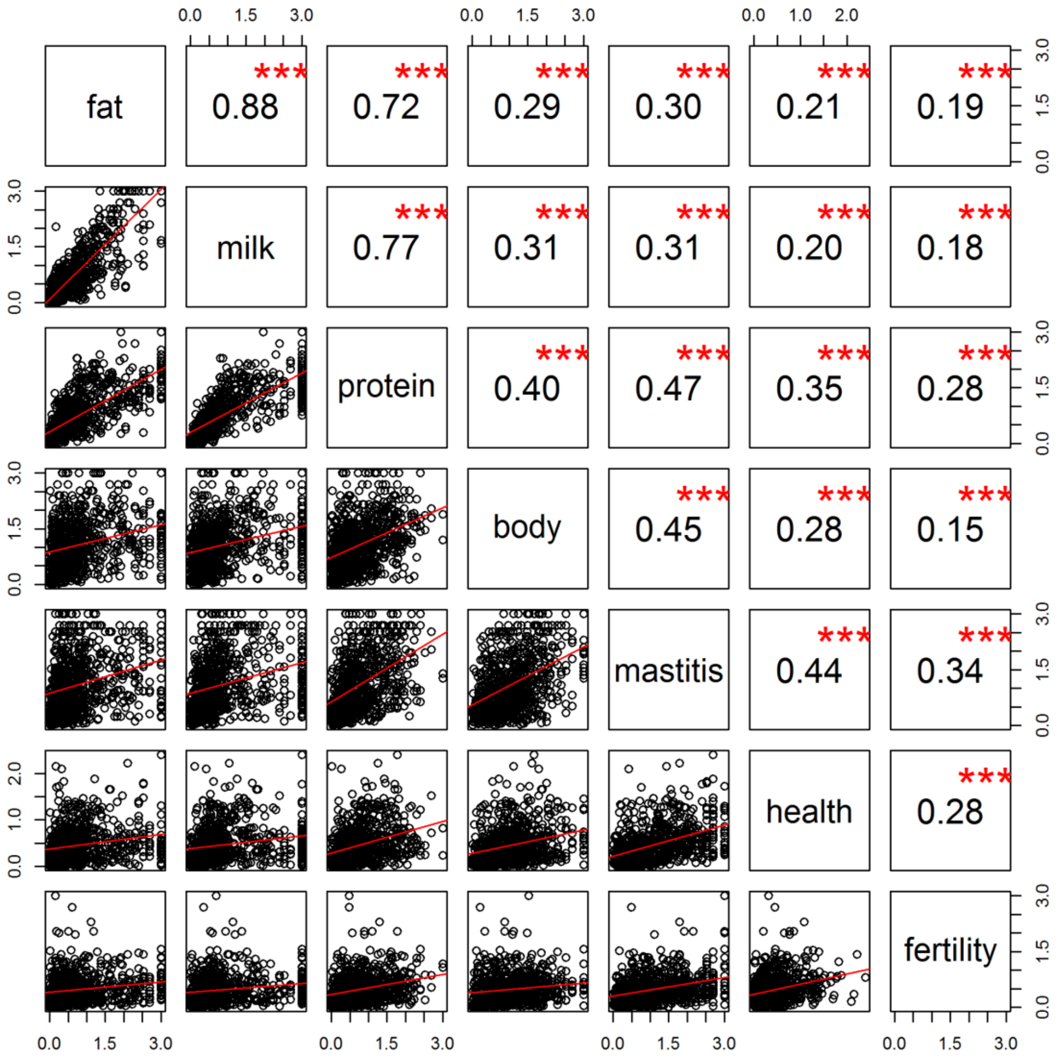

Supplementary Fig. S6. The correlations between traits based on marker-set test analyses of miRNA-target networks in Holstein (HOL)

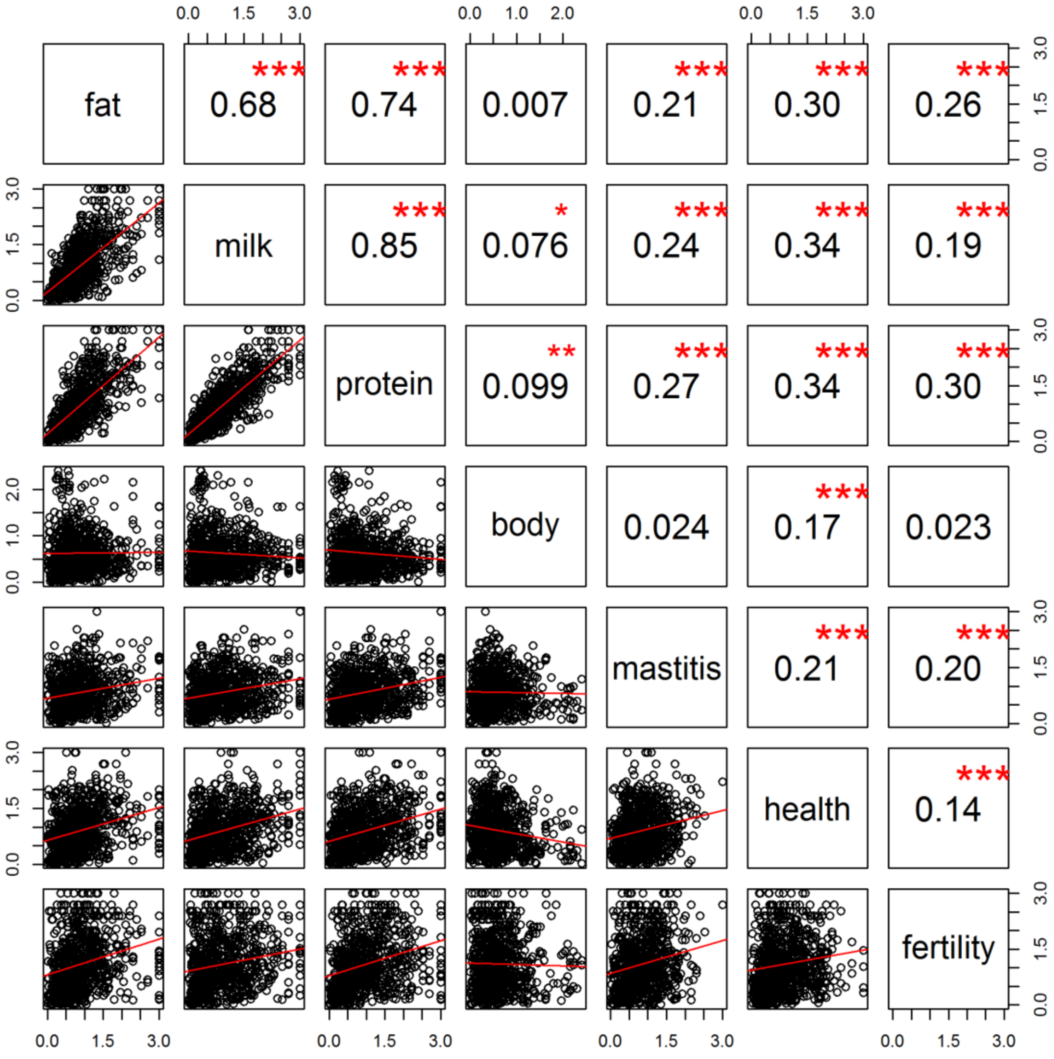

Supplementary Fig. S7. The correlations between traits based on marker-set test analyses of miRNA-target networks in Nordic red cattle (RDC)

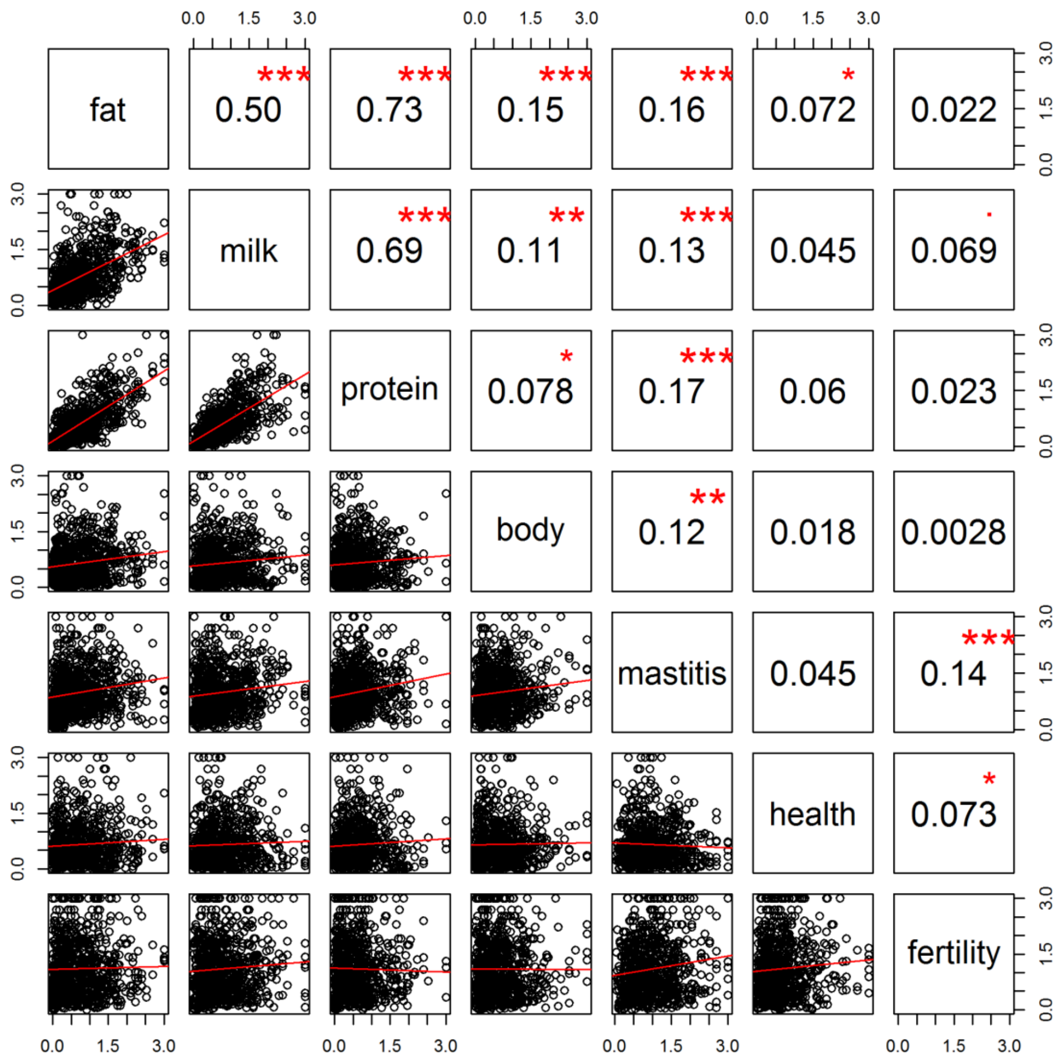

Supplementary Fig. S8. The correlations between traits based on marker-set test analyses of miRNA-target networks in Jersey (JER)

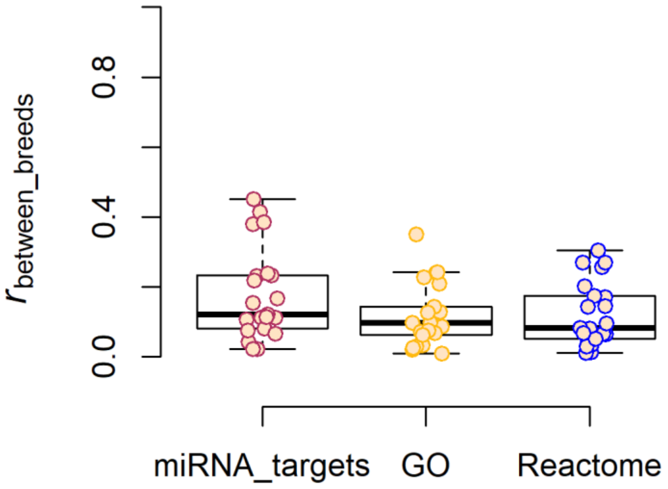

Supplementary Fig. S9. The difference in between-breeds correlations of enrichments among miRNA-target networks, Gene Ontology (GO) and Reactome terms

Supplementary Table S1. General characteristics of quantitative trait loci (QTL) detected in seven complex traits in three dairy cattle breeds using single-marker genome-wide association

**Trait**	**Breed**	**BTA**	**QTL regions**	**# of SNPs**	**Position (top SNP)**	**rsID (top SNP)**	***P*-value (top SNP)**	H^2_{snp}(%)	H^2_{total} (%)
**MY**	**HOL**	5	93944849-93948804	76	93944849	rs109422971	8.79e-16	2.2	**24.1**
		14	1569809-1892784	880	1825125	rs208113678	6.16e-86	13.9	
		20	29996719-34170153	26353	29996727	rs43116342	1.79e-12	2.3	
		23	17821120	1	17821120	rs472216679	1.55e-09	4.3	
		23	32417317-32417355	10	32417347	rs443822447	1.75e-09	0.2	
		26	36022272-44280829	66341	37869380	rs456998485	3.80e-15	1.2	

	**RDC**	5	112343204-112450860	700	112343204	rs383553819	2.00e-09	1.1	**13.0**
		14	1722033-1892559	441	1743939	rs133033480	9.75e-34	7.2	
		16	1322611	1	1322611	rs108979795	2.63e-09	0.1	
		19	61447138-61449096	10	61449096	rs210324693	1.19e-09	0.6	
		20	31909478-32647105	3904	31909478	rs385640152	2.78e-16	3.2	
		25	1627283-3516671	17017	3498960	rs110749311	4.83e-10	0.8	
	**JER**	14	1799066-1950961	366	1828456	rs135431494	1.21e-21	2.8	**3.9**
		20	33922713	1	33922713	rs440493451	3.38e-09	1.1	
**FY**	**HOL**	5	93945738-93954751	153	93945991	rs208248675	8.95e-26	1.8	**22.3**
		14	1706207-1823757	314	1810124	rs133931291	3.64e-132	18.3	
		26	20488043-21746946	6830	20547445	rs136702635	2.06e-22	1.5	
		29	17503000-25606561	80819	17696734	rs208845484	1.82e-10	0.7	
	**RDC**	5	93945694-93948804	59	93945694	rs209818856	3.23e-28	2.6	**13.3**
		14	1722033-1892559	441	1807140	rs136783505	9.80e-43	6.3	
		23	24986204-25169530	5172	24986204	rs516784604	2.46e-09	0.7	
		23	28567796-28591530	1093	28567796	rs381390819	4.35e-10	0.8	
		25	8663907-9997825	12325	9870005	rs379546164	3.98e-15	1.3	
		26	23948021-29821952	56815	24379571	rs438420348	5.20e-15	1.6	
	**JER**	14	1756075-1892559	59	1802667	NA	9.36e-15	3.1	**3.1**
**PY**	**HOL**	6	88477501-88481616	48	88478678	rs380681629	9.79e-10	1.5	**19.3**
		14	1448510-2080840	2133	1892784	rs207633422	6.93e-22	2.9	
		18	57015407-57038020	129	57015407	rs378839347	4.23e-11	1.2	
		23	10504197	1	10504197	rs379304770	4.80e-11	5.0	
		23	14536448-16488114	14330	14536448	rs382497921	6.89e-10	0.7	
		25	36371535-39391747	26269	36403719	rs135912796	8.36e-14	0.9	
		26	37695494-41579300	28401	41231611	rs136027702	4.72e-19	1.4	
		28	10749791	1	10749791	rs797640658	1.91e-09	1.9	
		29	12122106-14947666	30336	12741604	rs453104040	3.38e-10	2.6	

		29	17617875-17696775	471	17692377	rs137254498	6.09e-10	0.6	
		29	28915962-28966625	300	28915962	rs798193137	1.12e-09	0.7	
		29	35446115-35699001	2176	35699001	rs110014432	1.94e-09	0.6	
	**RDC**	5	112450860	1	112450860	rs109041054	1.32e-09	1.1	**9.3**
		14	1802667	1	1802667	NA	3.02e-09	1.1	
		23	8581891	1	8581891	NA	1.22e-09	0.8	
		25	294674-300548	53	298901	rs132704315	4.34e-10	0.9	
		25	3306363-3516671	2168	3498960	rs110749311	1.98e-12	1.0	
		26	10268885-10268923	5	10268885	rs449946672	4.48e-10	4.4	
**BC**	**RDC**	4	4495782-5368115	9752	4495782	rs208985165	5.01e-11	2.6	**33.3**
		5	105821790-106292827	2934	106252827	rs137527436	5.50e-11	1.9	
		6	38614055-39518299	6470	38651270	rs799919722	9.82e-71	22.4	
		14	24973953-24991209	75	24974811	rs137303549	7.86e-33	6.4	
**Mastitis**	**HOL**	6	88683517-89081567	5275	88840407	rs381295092	5.55e-19	4.1	**9.7**
		13	59258639-62017506	19057	59260175	rs42927066	2.09e-11	2.0	
		18	43909571-43924396	109	43909571	rs464881101	2.44e-10	1.9	
		23	9156030-13608649	34191	11477969	rs110729743	1.10e-13	1.3	
		25	35353527-37918904	23464	35353527	rs43722749	2.89e-10	0.4	
	**RDC**	6	88683517-89070689	5058	88723742	rs436532576	2.31e-10	2.3	**6.8**
		24	60,959,835	1	60959835	rs380418573	3.72e-10	4.5	
**Health**	**HOL**	6	88770377-89147179	5241	89118971	rs384330814	2.00e-11	8.2	**8.2**
**Fertility**	**HOL**	24	29556826-36261453	49258	34729148	rs451469340	1.19e-10	1.3	**5.3**
		24	61446132-61954906	3944	61552100	rs211207269	1.32e-10	4.0	
	**RDC**	12	20893092	1	20893092	rs384315836	3.62e-14	3.6	**8.1**
		25	325322	1	325322	rs109501400	1.90e-09	1.3	
		25	13063588-14584307	16170	13430119	rs380980565	3.00e-13	1.7	
		26	29797179-29821952	398	29797179	rs379654767	1.21e-09	1.5	

Supplementary Table S2. Number of miRNA genes (number of SNPs within miRNA genes) included in marker-set test based on different up- and down-stream extensions of miRNA genes (*i.e.*, 0kb, ±3kb, ±5kb, ±10kb, ±20kb, ±50kb)

**Breed**	**0kb**	**±3kb**	**±5kb**	**±10kb**	**±20kb**	**±50kb**
**HOL**	126(178)	657(17,320)	661(28,314)	665(55,122)	671(108,706)	680(272,061)
**RDC**	127(173)	658(18,051)	664(29,526)	666(57,614)	673(113,233)	682(282,179)
**JER**	106(160)	652(14,697)	658(24,013)	661(46,633)	667(91,895)	679(233,658)

Supplementary Table S3. Enrichments (*P* values, marker-set test) of miRNA genes including or excluding (in brackets) miRNAs close to *DGAT1* for three milk production traits

	**Fat yield**	**Milk yield**	**Protein yield**
**HOL**	0.001(0.106)	0.001(0.099)	0.040(0.082)
**RDC**	0.001(0.031)	0.030(0.024)	0.004(0.011)
**JER**	0.001(0.017)	0.003(0.001)	0.007(0.003)
